# Early Intervention for Spinal Cord Injury with Human Induced Pluripotent Stem Cells Oligodendrocyte Progenitors

**DOI:** 10.1371/journal.pone.0116933

**Published:** 2015-01-30

**Authors:** Angelo H. All, Payam Gharibani, Siddharth Gupta, Faith A. Bazley, Nikta Pashai, Bin-Kuan Chou, Sandeep Shah, Linda M. Resar, Linzhao Cheng, John D. Gearhart, Candace L. Kerr

**Affiliations:** 1 Department of Biomedical Engineering, Johns Hopkins University School of Medicine, Baltimore, Maryland, United States of America; 2 Graduate Program in Cellular and Molecular Medicine, Johns Hopkins University School of Medicine, Baltimore, Maryland, United States of America; 3 Institute for Cell Engineering, Johns Hopkins University School of Medicine, Baltimore, Maryland, United States of America; 4 Division of Hematology in Department of Medicine, Johns Hopkins University School of Medicine, Baltimore, Maryland, United States of America; 5 Department of Neurology, Johns Hopkins University School of Medicine, Baltimore, Maryland, United States of America; 6 Department of Obstetrics and Gynecology, Johns Hopkins University School of Medicine, Baltimore, Maryland, United States of America; 7 Department of Cell and Developmental Biology in the School of Medicine, University of Pennsylvania, Philadelphia, Pennsylvania, United States of America; 8 Department of Animal Biology in the School of Veterinary Medicine; University of Pennsylvania, Philadelphia, Pennsylvania, United States of America; 9 Department of Oncology, Johns Hopkins University School of Medicine, Baltimore, Maryland, United States of America; 10 Department of Pediatrics, Johns Hopkins University School of Medicine, Baltimore, Maryland, United States of America; 11 Singapore Institute for Neurotechnology, National University of Singapore, Singapore, Singapore; 12 Departments of Orthopedic Surgery, Biomedical Engineering and Medicine, Division of Neurology, National University of Singapore, Singapore, Singapore; 13 Department of Biochemistry and Molecular Biology, Unversity of Maryland School of Medicine, Baltimore, Maryland, United States of America; University of South Florida, UNITED STATES

## Abstract

Induced pluripotent stem (iPS) cells are at the forefront of research in regenerative medicine and are envisaged as a source for personalized tissue repair and cell replacement therapy. Here, we demonstrate for the first time that oligodendrocyte progenitors (OPs) can be derived from iPS cells generated using either an episomal, non-integrating plasmid approach or standard integrating retroviruses that survive and differentiate into mature oligodendrocytes after early transplantation into the injured spinal cord. The efficiency of OP differentiation in all 3 lines tested ranged from 40% to 60% of total cells, comparable to those derived from human embryonic stem cells. iPS cell lines derived using episomal vectors or retroviruses generated a similar number of early neural progenitors and glial progenitors while the episomal plasmid-derived iPS line generated more OPs expressing late markers O1 and RIP. Moreover, we discovered that iPS-derived OPs (iPS-OPs) engrafted 24 hours following a moderate contusive spinal cord injury (SCI) in rats survived for approximately two months and that more than 70% of the transplanted cells differentiated into mature oligodendrocytes that expressed myelin associated proteins. Transplanted OPs resulted in a significant increase in the number of myelinated axons in animals that received a transplantation 24 h after injury. In addition, nearly a 5-fold reduction in cavity size and reduced glial scarring was seen in iPS-treated groups compared to the control group, which was injected with heat-killed iPS-OPs. Although further investigation is needed to understand the mechanisms involved, these results provide evidence that patient-specific, iPS-derived OPs can survive for three months and improve behavioral assessment (BBB) after acute transplantation into SCI. This is significant as determining the time in which stem cells are injected after SCI may influence their survival and differentiation capacity.

## Introduction

Since the discovery of induced pluripotent stem (**iPS**) cells, the field of regenerative medicine has grown exponentially, and the feasibility of ‘adult cell-derived’ therapy is emerging. One of the main goals of iPS cell research is the derivation of stem cell lines that can be used to replace diseased or damaged tissues without generating a significant host immune response or relying on embryonic sources of cells [1–3]. A highly promising study by Wang et al. showed that human iPS OPs survived as long as 9 months following tissue grafts in the brains of shiverer mice, robustly myelinating axons and substantially increasing the survival rate of the mice [4]. However, the optimism regarding the use of iPS cells is tempered by concerns regarding their effectiveness for specific therapies, such as spinal cord injury (**SCI**).

A number of studies have investigated transplantation of oligodendrocyte progenitors (**OPs**) derived from human embryonic stem (**ES**) cells or mesenchymal stem cells (**MSCs**) in animal models of SCI, with some conflicting results. Previously, Yoshihara et al. reported that after transplantation of MSC in rats with SCI, there was no correlation between cell survival and locomotor improvement [5]. Yet more recently, Torres and Espín et al. published a promising study in which acutely grafted mesenchymal stromal cells in rat SCI led to improved locomotion [6]. Injections of bone marrow-derived MSCs have also been shown to improve hindlimb locomotion, reduce cavity area, and reduce inflammation in rats [7–9] and to improve recovery of the panniculus reflex and diminish pain responses in dogs with SCI [10].

The contradictions in the results of these studies include not only the efficiency of OP differentiation but also the time at which these cells are transplanted after injury. For instance, most studies have performed cell transplants one week or more after injury, after which the initial trauma to the spinal cord has already been compounded by secondary injury mechanisms, including glial scarring and cavitation at the injury epicenter [11,12]. The alternative is to perform acute transplantation of cells immediately following the injury. However, a concern for early cell transplantation of OPs is that the injured spinal cord environment would either kill or inhibit the differentiation of transplanted OPs. Given emerging evidence that progenitor cells respond to stress stimuli to facilitate tissue regeneration [13], it seems reasonable that OPs, unlike more mature cells, can survive the oxidative and immunological stresses of the injured spinal cord and that this may help facilitate their differentiation.

In similar fashion, early transplant studies with neural stem cells have demonstrated the ability of these progenitor cells to survive the hostile injured SCI environment and to provide neuroprotective effects that reduce secondary degeneration and preserve neuronal and oligodendrocyte survival [14–16]. For instance, work by Teng et al., demonstrates that animals implanted with a scaffold containing neural stem cells (**NSCs**) in hemisectioned spinal cords results in long-term improvements in motor function, related to a reduction of secondary degeneration, and the ability of NSCs to antagonize excitotoxic mechanisms [16]. Similar results were shown by human embryonic germ cell-derived embryoid body (**EB**)-derived cells in a virus-induced motor neuron disease model when EB-derived cells transplanted into the lumbar cerebrospinal fluid migrated to the damaged tissue and reduced motor neuron death [17]. Acute transplantation of MSC into injured spinal cord has also shown improved outcomes [6]. Recently, it was shown that a combined therapy of MSC-NSC intravenous injection immediately following SCI in rats lead to improved locomotion 6 weeks after injury, although there was no evidence of MSC survival beyond 2 weeks [18]. Studies directly compared early and delayed transplantation of MSCs have also revealed that only early-grafted cells improved locomotion and electrophysiological outcomes post-injury, although MSC grafts did not survive past 2 weeks [6]. Our group has also shown similar improvements using ES-derived OPs and extends this approach to iPS-derived OPs [19]. Thus, we hypothesized that iPS-OPs transplanted within 24 hours of injury can survive implantation, differentiate into mature oligodendrocytes, and integrate into the injured spinal cord.

This study is the first to describe how early transplantation of iPS-OPs is feasible for three independent iPS-derived cell lines, including two generated by integrating retroviral vectors (MR31 and A14) [20,21] and one by a non-integrating, episomal plasmid (BC1) [22]. Our results show that iPS-OPs reduce cavity formation, scarring and microglial proliferation in a rat model of moderate contusive SCI. The transplanted iPS-OPs survived up to the end of this two-month study, and by this time roughly half of the transplanted OPs expressed the mature oligodendrocyte marker, myelin basic protein (**MBP**). We further show that transplanted cells lead to increased remyelination and/or reduced demyelination of axons at the epicenter of injury, evidence that early intervention using iPS-OPs is feasible for reducing SCI pathology. In addition, we provide a thorough characterization of the differentiation of iPS-OPs, which has not been previously reported. This knowledge provides a foundation for future studies whose goals are to follow or understand how iPS cells differentiate into OPs as well as to study the optimal cells and media conditions for OP regenerative therapies.

## Materials and Methods

### Ethics

The experimental design using human iPS cells was approved by the Institutional Stem Cell Research Oversight (ISCRO) committee in the Johns Hopkins University. Use of anonymous human samples for laboratory research was approved by JHU Institutional Review Board on Human subjects (ESC-09-05-20-01). This included the acquisition of CD34+ cells for iPS cell generation from adult patients [20,22]. Fetal brain samples that were used as a reference for qRT-PCR analysis was supplied by the University of Washington NIH-supported fetal repository. In vivo study was carried out in accordance with the guidelines of the Johns Hopkins University for the Care and Use of the Laboratory Animals. The protocol was approved by the Committee on the Ethics of Animal Experiments with the Protocol Number: RA12M82.

### Cell Culture

Three different iPS cell lines were used in this study: BC1 [22], MR31 [21] and A1-4 [20]. The sources of the iPS cells and the reprogramming methods used are summarized in S1 Table. The BC1 iPS cell line was generated from adult CD34+ peripheral blood monocytes using an episomal plasmid (pEB-C5) expressing 5 reprogramming vectors (*OCT4*, *SOX2*, *KLF4*, *MYC*, *LIN28*). Notably, whole-genome sequencing of the BC1 iPS line generated using an episomal plasmid vector revealed that the vector sequence was undetectable in the genome [22,23]. A1-4 was generated by reprogramming MSCs with retroviruses expressing *OCT4*, *SOX2*, *KLF4*, *MYC*, and the high mobility group A1 chromatin remodeling protein, HMGA1 [20]. MR31 was generated from the IMR90 fetal lung fibroblast cell line reprogrammed with retroviruses expressing *OCT4*, *SOX2*, and *KLF4* [23]. iPS cells were cultured and differentiated into OPs as described previously [19,22,24,25].

Undifferentiated iPS cells were propagated on a mouse embryonic fibroblast (**MEF**) feeder layer. All lines were expanded in iPS cell growth media containing DMEM/F12 supplemented with 20% knockout serum, 5% nonessential amino acids, 5% Glutamax, and 20 ng/ml Fibroblast Growth Factor 2 (FGF2) [22]. The iPS cells were differentiated to OPs using methods optimized by our laboratory for ES cells [24–26]. First, neural differentiation of undifferentiated iPS cells was initiated by embryoid body (**EB**) formation in neurobasal media (Gibco) supplemented with Noggin and FGF2 (200 ng/ml and 20 ng/ml, respectively; R&D Systems). EBs were maintained in ultra-low adhesion tissue culture plate for 15 days. EBs were plated onto matrigel-coated tissue culture plates in neurobasal media supplemented with FGF2 (20 ng/ml) for 5 days, leading to differentiation into neural progenitors (**NPs**). NPs were expanded in this media for 5–7 days and glial progenitors (**GPs**) were subsequently induced by adding EGF (10 ng/ml; R&D Systems). NPs and GPs are distinguished by their morphology, marker expression and their differentiation capacity. Our laboratory has shown that NPs are able to differentiate into neurons and glial cells, while GPs primarily develop into either astrocytes or oligodendrocytes that are regulated by specific cell culture requirements [26,27]. After 7 days in GP media, cells are split with 1 mg/ml collagenase and transferred into new plates. OPs were induced by adding neurobasal medium supplemented with EGF (20 ng/ml) and PDGF-AA (20 ng/ml; Peprotech) for 25 days. To determine differentiation efficiency among iPS lines, cells were harvested and RNA extracted for quantitative RT-PCR (qRT-PCR) analysis or cells placed in chamber slides for immunocytochemistry and protein expression analysis. A subset of OP cultures were also sorted by O4 expression using magnetically-activated cell sorting (MACs) and further expanded in culture for use in cell transplantation. O4 is a sulfated galactosylceramide (sulfolipid) synthesized primarily by oligodendrocytes and their progenitors OPs that has been shown to play a major role in myelin function and stability [28]. O4 expression is also expressed by NG+ OPs in the human fetal brain [29]. Using this method, we are able to maintain and expand cultures containing over 98% O4+ OPs that proliferative for up to six months in culture.

**Immunocytochemistrical Characterization and Cell Counting.** Cells were plated onto chamber slides. Before they became confluent, cells were washed twice in phosphate buffered saline (PBS) and the excess PBS removed. Cells were then fixed in 4% paraformaldehyde (PFA) in PBS for 10 minutes at room temperature (RT). The fixative solution was removed and cells were washed twice with PBS for 5 min each. Cells were permeabilized and blocked with PBS solution containing 0.2% (w/v) Triton X-100 (Sigma) and 1% (w/v) bovine serum albumin (BSA; Sigma) for 5 minutes at room temperature (for cell surface markers this step was omitted as previously described by [29]. Primary antibody (1:50 dilution, see S2 Table for list of the primary antibodies) was added after dilution in antibody dilution buffer (PBS with 0.1% (w/v) BSA) and incubated for 1 hr at RT. Samples were washed three times with PBS for 5 minutes each. Appropriate secondary antibodies were added (Alexa 488 or Alexa 564 conjugated antibodies, 1:200; Molecular Probes) in antibody dilution buffer and incubated in a humidified dark chamber for 1 hr at RT. Cells were then washed three times with PBS for 5 minutes each. Nuclei were stained using 4',6-diamidino-2-phenylindole (**DAPI**) solution (Sigma) for 10 min at RT in a humidified chamber in the dark. DAPI solution was aspirated and three 0.5 ml PBS rinses were performed and slides coverslipped. Differentiation of each cell line was performed in triplicate. Cells exposed to secondary antibody alone were used as negative controls. The number of positively stained cells were counted using Metamorph Image Analysis Software (Universal Imaging, Inc.) from 3 randomly, chosen 10X fields per 24-well cell culture dish and each stain was repeated in three wells (n = 9).

**Quantitative Real Time-PCR Analysis.** Total RNA was isolated from cells stored in RNAlater (Qiagen) solution using MiniRNeasy kits (Qiagen 74124) and complementary DNA (cDNA) generated with SuperScript III First-Strand Synthesis System (Invitrogen 18080–051) reverse transcription kits following the manufacturer instructions. Real-time quantitative polymerase chain reaction (qPCR) analyses were performed using ABi7900HT (ABiosystems) in 96 well plates (Abi N801-0560) where each real-time amplification had a template equivalent to 5 ng of total RNA. Each primer set was tested in at least triplicate across 3 biological replicates. Negative controls included a reverse-transcription-negative control per sample and a no template control. For these analyses, total RNA extracted from a 92-day old fetal human brain was used as a positive control. Gene expression and amplification curves were normalized to β-actin. Taqman Assay-on-Demand designed oligonucleotides were used for the detection of the following genes (S3 Table). Using the ∆Ct method, quantification within the log-linear phase of the amplification curve acquired for each probe/primer set was normalized to β-actin and plotted.

### Contusive Spinal Cord Injury

All experimental procedures in this study adhered to the guidelines delineated in the *Rodent Survival Surgery* guide and approved by the Institutional Animal Care and Use Committees at the Johns Hopkins University. An ARRIVE Guidelines Checklist for reporting in vivo experiments is provided in Supporting Information (S1 Checklist). A total of 63 rats were used in the following study, twelve rats were injected with either BC1-, MR31-, or A1-4-derived OPs, PBS alone or heat-killed BC1-derived OPs and 3 rats with laminectomy alone (sham) were used for comparisons with electron microscopy. Ten-week-old adult female Lewis rats weighing ~200–220g were used in the study. One of the immediate side-effects of SCI is difficulty urinating and thus the bladder of these rats must be manually expressed for days immediately after injury and are thus susceptible to infection. Therefore, this study utilized female rats which are less suspectible to urinary tract infections under these conditions [30]. All rats were immunocompromised three days prior to cell transplantation followed by daily injections of cyclosporine A (10mg/kg/d) until the end of the study, as previously described [24]. Rats (n = 12 per treatment) were anesthetized with ketamine (7.5 mg/kg; Phoenix Pharmaceutical) and xylazine (60 mg/kg; Phoenix Pharmaceutical). Laminectomy was performed at thoracic vertebra T8 without opening the dura mater. Injury was produced using a Multicenter Animal Spinal Cord Injury Study (MASCIS) Impactor, formerly called the NYU-Impactor. The MASCIS impactor model is a well-established model for studying contusive SCI in rats, and histopathological changes closely mirror those observed in human SCI [31,32]. This device precisely measures the movement of a 10 gram rod dropped from 6.25, 12.5, 25.0, or 50.0 mm heights onto a rat T8-T10 spinal cord after laminectomy to induce Mild (6.25 mm), Moderate (12.5 mm), Severe (25.0 mm) and Very Severe injury (50 mm) respectively. In this report, the MASCIS impactor consisting of a 10.0 g weight rod dropped from a 12.5 mm height was utilized to produce a moderate contusion injury, as previously described [33–37].

**Cell Transplantation.** Rats were anesthetized 24 hours after injury as described above, and the injury site on the spinal cord was exposed and the dura excised using a 30-gauge needle. O4+ OPs cultured in OP-inducing media for 25 days were used as the majority of cells (> than 98%) expressed OP marker O4. Viability was also assessed by sampling a subset of cells with 0.4% trypan blue (Sigma) prior to transplantation and only populations with at least 90% viability were transplanted except for heat-killed controls which had their membranes disrupted by heating samples at 65°C for 90 minute followed by sonication.

Twelve rats were randomly selected and injected with either 500,000 live iPS-OPs or heat-killed iPS-OPs in 2 μL volumes with PBS at a rate of 1 μL/minute into the posterior funiculi of T8 at a depth of 1.2 mm. This region is known as the injury epicenter. Cells were injected using a 10 μL Hamilton Gastight syringe with an attached borosillate glass tip (50–100 μm tip diameter) secured to a manual micromanipulator (World Precision Instruments) adjusted with a 80° tilting base. Briefly, the tip is lowered to a depth of 1.2 mm below the surface of the cord and is held in place for 2 minutes before the cell injection and 2 minutes after the cell injection. Cells are delivered under the control of a micro-syringe pump controller (World Precision Instruments). After injection, the dura is closed with a 9–0 suture, the muscles re-apposed, and the skin closed with sutures. A suture is also placed in the muscle adjacent to the site of injection to locate the injection site at the time of sacrifice. Sites are monitored for bleeding for the next six hours and for infections over the next five to seven days. Rats were housed two per cage in a pathogen-free isolation unit and given subcutaneous injections of isotonic saline (20 ml/kg), which was administered every morning for 7 days after surgery. The rats’ bladders were expressed twice daily, and the rats were inspected for weight loss, discomfort, dehydration, and autophagia, with appropriate veterinary care as needed. Tylenol (100 mg/kg) and gentamicin (5 mg/kg) were administered every morning for 7 days to minimize pain and potential for infection, respectively. In addition to these study groups, three additional uninjured rats were employed and served as sham controls (undergoing laminectomies only) to compare the extent of myelination in the injured SCIs after transplant. These rats were treated under identical conditions following surgery.

**Motor Behavior Assessment.** The Basso, Beattie, and Bresnahan (BBB) Locomotor Rating Scale [38,39] was used to validate the motor behavior of each animal before and after injury. Two well-trained examiners, who were blinded to the experimental group of the animals, evaluated and scored the motor behavior of each animal for the left and right sides individually. The left and right sides were averaged. Scoring was performed on each animal every three days for the first four weeks, and thereafter performed once per week.

**Histological Analyses.** Postmortem characterization of transplants was observed up to two months after transplantation. Rats were deeply anesthetized using isoflurane. Transcardial perfusion was performed with DPBS (Gibco) and paraformaldehyde (PFA) solution (4%; 15713-S, Electron Microscopy Sciences). Spinal cords from twelve rats within each group were randomly prepared for either frozen sectioning, paraffin embedding or prepared for electron microscopy and histological assessments were performed by a laboratory technician who was blinded to the experimental groups. Thus, four rats of each treatment group were either prepared for cryosection, paraffin sectioning, or embedded for electron microscopy. The spinal cord was then carefully extracted from the vertebrae column and post-fixed in 4% PFA, followed by 30% sucrose solution for 24 hours, and last embedded in paraffin or cryopreserved in freezing compound (O.C.T. Tissue-Tek). Frozen tissue was cut in 5 μm sections and prepared for immunohistochemistry. For analysis of frozen sections, 30 histological sections, sliced 5μm thick were analyzed that encompassed the injury epicenter. Immunoflourescent staining with different antibodies was performed on adjacent slices for each spinal cord. Immunostaining was performed by incubation with 1:100 dilution of primary antibodies (S2 Table) overnight at 4°C in 15% FBS, followed by three rinses in DPBS, and incubated with 1:500 dilution of Alexa 488 and Alexa 564 secondary antibodies in 15% FBS for 1 hour at room temperature and coverslipped with DAPI-supplemented mounting media (Vectashield by Vector Labs). DAPI was used to stain nuclei and to determine the percent of immuno-positive cells. Negative controls included primary serum and secondary antibody alone. Cords embedded in paraffin were cut in 10 μm longitudinal sections and stained with either hematoxylin and eosin (H&E) or luxol fast blue (LFB). Sections encompassing the injury epicenter and surrounding areas of injury were selected by consecutively numbering each cut beginning from the dorsal surface. and then every other slice was stained with either LFB or H&E. The demarcation of glial scarring and cavitation in controls allowed for easy assessment to determine the boundaries around the epicenter of injury. This allowed the ability to analyze similar numbers of cuts across treatment groups. To analyze staining of images across groups were taken at the same exposure time below the level of saturation and analyzed within the same gray scale. The optical intensities was measured in identically-sized fields surrounding the injury epicenter and background subtracted from a region on the same slide surrounding the tissue using Metamorph Image Analysis Software.

**Survival and Differentiation of Ops.** To determine whether injected human iPS-OPs could be detected at the end of the study, frozen sections were analyzed using the human cell-specific marker, human nuclear antigen HNA (Millipore) to determine the number of human cells present. To determine the number of OPs that differentiated into mature oligodendrocytes, we counted the number of HNA/DAPI-positive cells that showed co-localized staining with myelin basic protein (MBP), a protein specifically expressed by mature oligodendrocytes and not by OPs. To study how OP transplantation may alter the immune response after SCI, sections were stained for CD68, (also known as ED-1) to count the number of immune cells (macrophages, microglia and T-cells) located at the injury epicenter [40]. Astrocyte response and glial scarring was also quantified by studying the number of GFAP-expressing cells at the epicenter of injury and by the fluorescence staining intensity around the cavity, as previously described [41].

**Tissue Sparing Analysis.** Images were analyzed using Metamorph Image Analysis Software, which allowed the tracing of the spinal cord perimeter and the central cavity for each image captured, giving the total area for each of these measurements. This analysis was performed on longitudinal spinal cord sections generated from 8 mm rostral to 8 mm caudal to the injury epicenter and encompassing the entire width of the spinal cord, as the cavity very rarely extended beyond this distance. Each cord was analyzed using 10 μm sections stained with hematoxylin and eosin and photographed at 16X magnification.

**Electron Microscopy.** For electron microscopy, spinal cords from 4 rats from each treatment group were processed into small 3 mm^3^ blocks that surrounded the injury epicenter and fixed for 1 hour in a mixture of glutaraldehyde (1.5%) and paraformaldehyde (3%), followed by washing three times in 0.1 M sodium cacodylate and 3 mM CaCl_2_. Samples were then post-fixed in potassium ferrocyanide (0.8%) and osmium tetroxide (1%) for 1 hour followed by 3X washes in 0.1 M sodium cacodylate, and 3 mM CaCl_2_. Following a brief dH_2_O rinse, samples were embedded in Eponate 12 (Pella), and cured at 60°C for 2 days. Spinal cord sections (80 nm in thickness) corresponding to the site of the lesion were cut on a Riechert Ultracut E with a Diatome diamond knife, collected on formvar-coated 1 X 2 mm^2^ copper grids, and stained with uranyl acetate followed by lead citrate. Sections were examined on a Hitachi 7600 transmission electron microscope (TEM) operating at 80 kV.

Myelination was examined on TEM sections at 10000X magnification. Normal myelinated, demyelinated, and remyelinated axons were counted using the line-sampling technique detailed by Blight [42]. The average number of normal myelinated, demyelinated, and remyelinated axons within five 25x25 μm^2^ areas along the radial line yielded an estimate of the total number of axons within the injury epicenter, calculated as the number of axons per square millimeter. At least 10% of the area of injury is encompassed in these calculations. The number of remyelinated and demyelinated axons was used to determine the oligodendrocyte remyelination efficiency.

### Statistical Analysis

All data were expressed as the mean ± SEM. Statistical significance was determined between treatment groups for cell counting and histological analyses by one-way ANOVA combined with Tukey’s posthoc test for comparison between groups. Similarly, significant differences in BBB scores among groups were also evaluated using a one-way ANOVA followed by a two-way Student’s t-test assuming equal variances.

## Results

### Morphological Characterization

**Fig 1:**
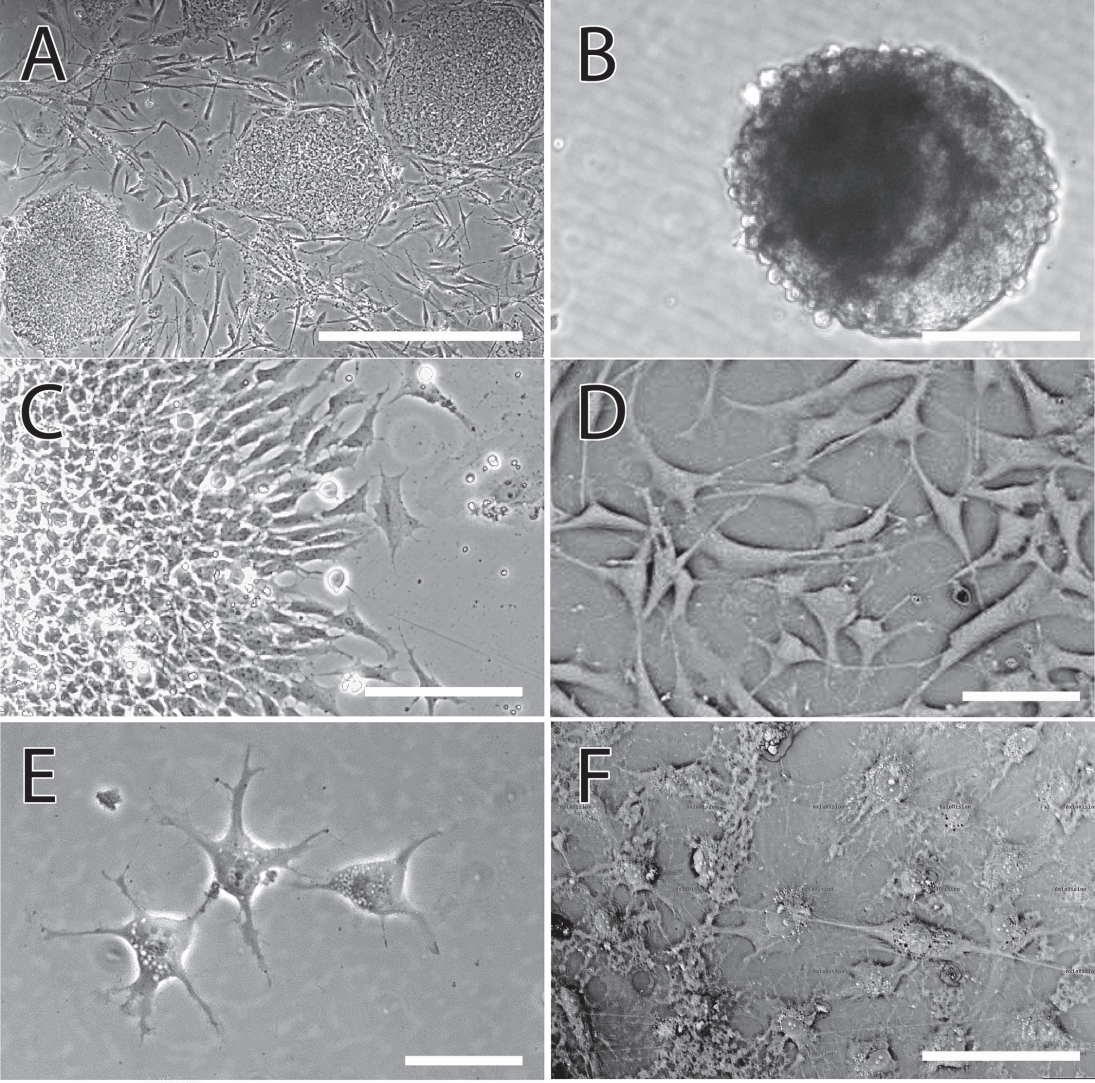
Bright field microscopy images of the various stages of oligodendrocyte differentiation from induced pluripotent stem cells of the BC1 line. (A) The undifferentiated cells grew in colonies on a mouse embryonic fibroblast feeder layer, (B) Embroid bodies (EBs) were formed by suspending the undifferentiating cells in culture on ultra-low adherence plates, (C) 15-day EBs were plated on matrigel, leading to the extension of cells of neuroectodermal lineage. Upon passaging, these neural progenitors grew in a monolayer and were subsequently differentiated to glial progenitors (D) stage. Addition of platelet derived growth factor-AA (PDGF-AA) to the media led to glial progenitors (GPs) transitioning into the (E) oligodendrocyte progenitor (OP) stage, displaying a rounded morphology with multiple filopodial extensions. (F) Terminal differentiation was induced by the removal of PDGF-AA, leading to the appearance of mature oligodendrocytes displaying characteristic branching. Scale bars- A-B: 300 μm, C-F: 80 μm

We derived OPs from three different human iPS cell lines using our published protocol to generate OPs from human ES cells [19,24]. We found that all three iPS cell lines exhibited morphologic changes consistent with a transition from the undifferentiated pluripotent stage into mature oligodendrocytes (Fig. 1). The transition from pluripotency into neuroectoderm was initiated by formation of embryoid bodies (EBs) in neurabasal media under low attachment conditions (Fig. 1B). EBs were cultured over a 15-day period and were then plated on matrigel in neurobasal media supplemented with FGF2 (20 ng/ml). Within 24 hours, cells with a neuroectodermal morphology were observed with projections extending outwards from the center of the EBs (Fig. 1C) indicative of neural progenitors (NPs) in culture. Adding EGF to the media led the cells to differentiate into glial progenitors (GP) with a bipolar morphology (Fig. 1D) [43]. At this stage GPs are bipotent and depending on the culturing media can be induced to become either OPs or astrocytes [26]. For transplantation studies, GPs were induced to differentiate into OPs by the addition of PDGF-AA and the removal of FGF2 from the media. The resulting cells had the characteristic rounded shape with multiple filopodial-like extensions (Fig. 1E). Upon removal of PDGF-AA from the culture media and with addition of 40 ng/ml 3,395-Triido-L-thyronine (T3), iPS-OPs demonstrate the ability to generate mature oligodendrocytes exhibiting branched morphology with high purity (> than 70%) in unsorted iPS-OP cultures (Fig. 1F).

### Quantitative Gene Expression Analysis

**Fig 2:**
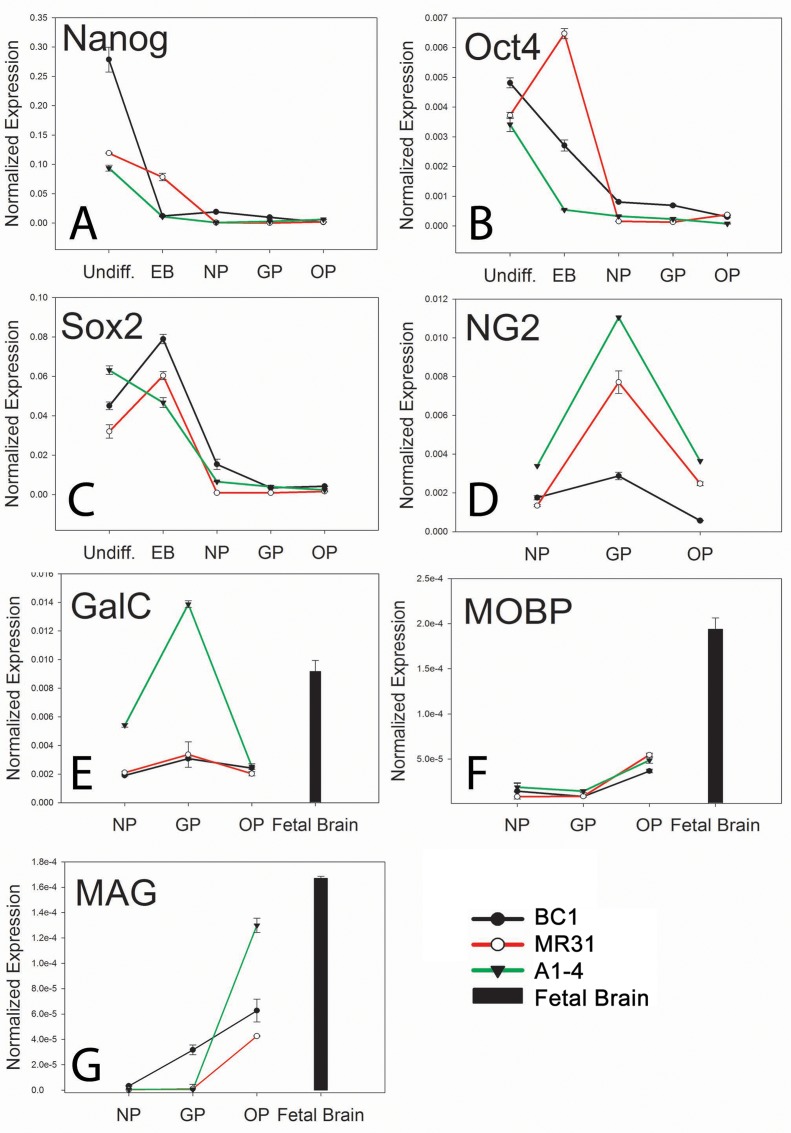
Plots showing mRNA expression of various developmental markers for all three iPS lines at each stage ofoligodendrocyte differentiation, as measured using qRT-PCR and normalized to fetal fibroblast controls. Expression of pluripotency associated genes (A) *NANOG*, (B) *Octamer-binding transcription factor 4 (OCT4)* also known as *POU5F1 (POU domain*, *class 5*, *transcription factor 1)* and (C) *SRY (sex determining region Y)-box 2 (SOX2) were undetected in oligodendrocyte progenitors (OPs)* whilemarkers indicative of the oligodendrocyte lineage were induced including (D) *NG2*, (E) *Myelin-associated glycoprotein* (*MAG)*, and (F) *Myelin-associated oligodendrocytic basic protein* (*MOBP)*. Delta CTs were calculated using β-actin as a relative standard and lines normalized to fetal fibroblast controls. Mean + SEM were calculated from two independent differentiation experiments for each line and each gene performed in technical triplicate.Positive control included total RNA from human fetal brain. Abbreviations are Undiff: Undifferentiated iPS cells, EB: embryoid body, NP: neural progenitor, GP: glial progenitor, OP: oligodendrocyte progenitor.

Quantitative real-time PCR (qRT-PCR) analysis was carried out to compare gene expression profiles in the cells as they progressed from a pluripotent state into OPs. To determine if the differentiated cells decrease expression of genes associated with pluripotency, mRNA expression levels of *NANOG*, *OCT4* and *SOX2* were analyzed (Fig. 2A-C). We found that the expression levels of these genes were significantly decreased or undetectable once cells reached the NP stage. Interestingly, *OCT4* expression showed a significant increase in the retrovirally-derived MR31 cell line at the embryoid body stage as compared to undifferentiated cells. These results suggest a persistence of *OCT4* expression in this cell line that remains undifferentiated under EB conditions. Nonetheless, *OCT4* transcript levels decreased after EBs were cultured as NPs. *SOX2* expression also increased in EBs of MR31 and BC1. This is not surprising as *SOX2* plays an essential role in early neuroectodermal differentiation [44] in addition to pluripotency. *NANOG* expression diminished during OP differentiation in all three lines. Most importantly, NP cultures from all three lines demonstrated significantly low or undetectable levels of *NANOG*, *SOX2* and *OCT4* mRNA transcripts, which is essential in preventing teratoma formation *in vivo*.

In addition to downregulating pluripotent genes, NPs also exhibited upregulated markers of early neural differentiation. For instance, *NG2* is expressed in both GPs and OPs found in fetal and adult spinal cord [29,45]. While undetectable in undifferentiated iPS, low levels were initially detected in NPs but significant rose 5 days after adding GP induction media in all three iPS-derived NP lines (Fig. 2D). The increase in *NG2* expression was more pronounced for the MR31 and A1-4 lines (both of which were derived from retroviral delivery of pluripotency factors), as compared to the BC1 line. However, the differences in *NG2* expression in GPs were transient, as *NG2* transcript levels decreased sharply in OPs where mRNA levels were similar in all three lines (Fig. 2D). To further confirm the identity of the OPs, we examined expression of genes encoding the myelin oligodendrocyte basic protein (MOBP) and myelin associated glycoprotein (MAG). Both of these proteins are expressed by myelinating oligodendrocytes [46,47]. As a positive control, total RNA was extracted from a 92-day old fetal human brain, at which time PDGFR-α +/O4+ OPs are found in the cortical subventricular zone [29]. We found that *MAG* expression was induced at the OP stage in all three lines (Fig. 2E). In fact, there was an almost linear increase in *MAG* expression as the cells progressed from the NP to OP stage in BC1. *MAG* expression was also relatively low in the MR31 and A1-4 lines at the GP stage, but rose sharply during differentiation to the OP stage. The *MOBP* transcript levels were also induced in all three OP lines (Fig. 2F), with negligible expression at the NP and GP stages and up-regulation at the OP stage. Expression of *MAG* and *MOBP* was higher in the control fetal brain tissue isolated at 13.3 weeks in gestation. This is consistent with other reports that have shown the presence of myelin marker expression as early as 5 weeks in gestation [29].

### Immunocytochemistry and Protein Marker Expression

**Fig 3:**
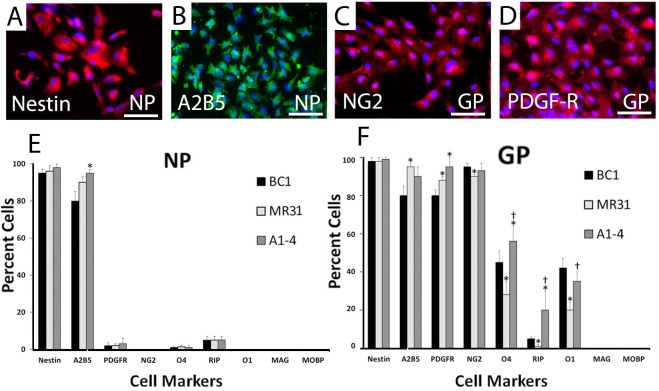
Indirect immunofluorescence detection of neural markers help define the initial stages of oligodendrocyte differentiation from undifferentiated iPS cells, into neural (NP) and glial (GP) progenitors. (A-D) Photos are of cells derived from BC1 with the differentiated stage (underlined) and marker analyzed as indicated similar staining patterns were seen in all three lines. Nuclei were stained using DAPI (blue). Most neural progenitors (NPs) were (A) NESTIN+ and (B) A2B5+ while the majority of glial progenitors (GPs) began expressing early oligodendrocyte progenitor (OP) markers (C) platelet derived growth factor receptor-α (PDGFR-α) and (D) NG2. Lower panel shows quantification of the indirect immunofluorescence analyses calculating the percent of labeled cells in all markers. (E) 90% of all cells from each line expressed early markers of the neural lineage including NESTIN and A2B5. (F) Likewise the number of glial progenitors expressing markers of the early OP lineage such as PDGFR-α and NG2 were also similar among lines. Differentiation of each cell line was performed in triplicate and the percent of positively stained cells was determined from 3 randomly, chosen 10X fields per 24-well cell culture dish from all three replicates. Values in the graph represent mean ± SEM. * and † denote statistical significance compared to BC1 and MR31, respectively by ANOVA and Tukey post hoc tests (N = 9, P < 0.001). Scale bars: 100 μm.

To corroborate the results for mRNA analyses, we also confirmed protein expression using immunofluorescence microscopy. Prior studies of oligodendrocyte development show that the first step in OP development is the differentiation of A2B5+ and NESTIN+ NPs into glial progenitors that express NG2 and PDGFR-α [24]. We also observed robust A2B5 and NESTIN protein expression in the NPs derived from EBs (Fig. 3A-B). As these cells differentiate into the GP lineage, they gain NG2 and PDGFR-α protein expression (Fig. 3C-D). Quantitative analysis was also performed on immunofluorescence images for three independent experiments for each line at each stage of differentiation. Quantitative analysis showed that >80% of the NPs were A2B5+ and NESTIN+ (Fig. 3E). In contrast, as expected, proteins associated with more differentiated OPs such as the RIP antigen, which specifically recognizes 2',3'-cyclic nucleotide 3'-phosphodiesterase in OPs and mature oligodendrocytes [48] as well as O1, MAG and MOBP, were low or undetected in NPs. In addition to NESTIN and A2B5 expression, greater than 80% of GPs in all three lines acquired NG2 and PDGFR-α expression (Fig. 3F). Moreover, some GPs also began expressing O4, RIP and O1 while MAG and MOBP expression was not detected.

**Fig 4:**
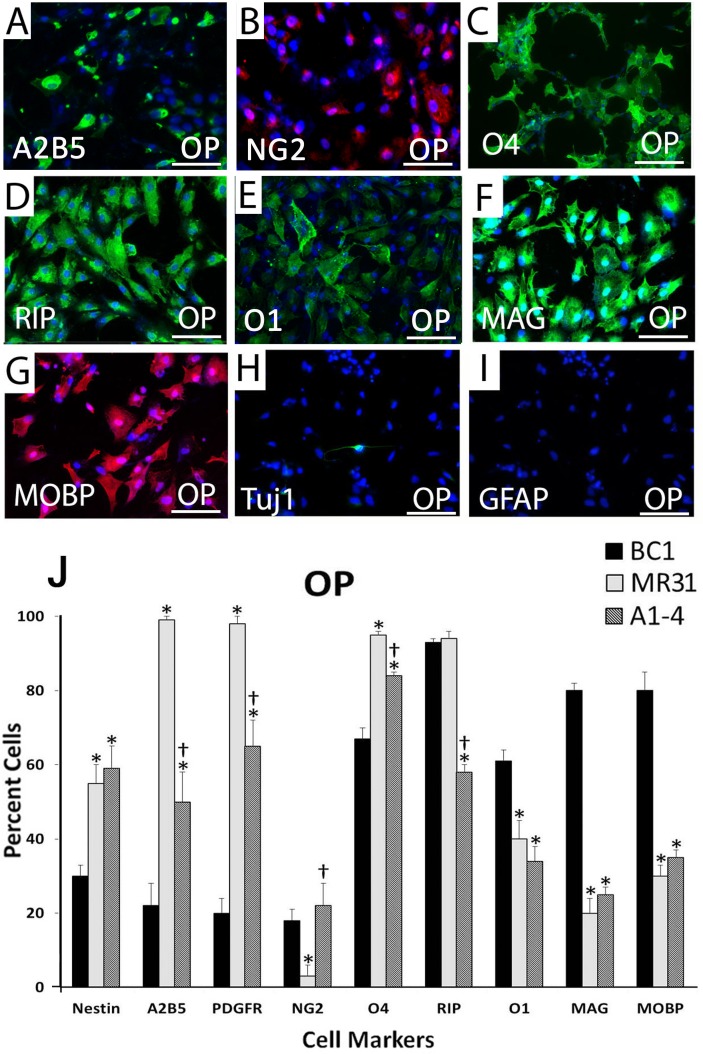
Indirect immunofluorescence marker characterization of oligodendrocyte progenitors derived from human iPS cells. Nuclei were stained with DAPI (blue). Results show reduced expression of early markers (A) A2B5 and (B) NG2 and increased protein expression of mid to late oligodendrocyte progenitor (OP) differentiation markers (C) O4 (D) RIP (E) O1 (F) myelin-associated glycoprotein (MAG) and (G) myelin-associated oligodendrocytic basic protein (MOBP). Myelin basic protein (MBP) expression was not detectable by immunostaining. 1% or less of OP cells expressed markers of other neural lineages as denoted by (H) the neuronal maker neuron-specific class III beta-tubulin (TUJ1) and (I) the astrocyte marker glial fibrillary acidic protein (GFAP). (J) Quantification of the indirect immunofluorescence analyses calculating the percent of cells expressing a particular marker is shown in the lower panel. A higher percent of cells derived from BC1 expressed later OP markers MAG, MOBP and O1 while fewer cells from the BC1 lines expressed early neural progenitor (NP) and glial progenitor (GP) markers and early OP marker O4 compared to MR31 and A1-4. Differentiation of each cell line was performed in triplicate and the percent of positively stained cells was determined from 3 randomly, chosen 10X fields per 24-well cell culture dish from all three replicates. Values in the graphs represent mean ± SEM. * and † denote statistical significance compared to BC1 and MR31, respectively by ANOVA and Tukey post hoc tests (N = 9, P < 0.001). Scale bars: 100 μm.

Differentiation into OPs was confirmed by proteins expressed in mid- to late-OP differentiation including RIP, O4, and O1 with downregulation of A2B5 and NESTIN staining. O1 is a marker of late OP differentiation, while O4 is expressed earlier [19]. These proteins were expressed in the majority of OPs derived from all three iPS cell lines (Fig. 4A-E). In addition to markers of early OP differentiation, iPS-OPs also expressed detectable levels of proteins associated with myelin and are used to identify mature oligodendrocytes that are capable of generating myelin, such as the MAG and MOBP proteins (Fig. 4F and 4G, respectively). Quantitative analyses also showed that the number of cells expressing O1 was higher than the number of OPs expressing O4 from the BC1 line (episomal, non-integrating plasmid delivery or iPS factors), which could indicate that BC1 derived iPS-OPs are more efficiently differentiated. In contrast, the A1-4 and MR31 lines had higher levels of O4, a marker of less differentiated OPs compared to O1. These proteins were not detected in the NPs or GPs (Fig. 3E). Of note, the neuronal marker, Neuronal Class III βTubulin (TUJ-1) and the astrocyte marker, glial fibrillary acidic protein (GFAP), were expressed in less than 1% of the cell population in all three iPS cells (Fig. 4H and 4I, respectively).

The more differentiated state of the majority of BC1-derived OPs is further supported by reduced NP marker expression such as NESTIN and A2B5, which were expressed by nearly twice as many cells in OP induction media (~60%) in the A1-4 and MR31 lines compared to BC1 OP cultures (~30%). Furthermore, more cells from the BC1-derived OP cultures expressed myelin-associated proteins, MAG and MOBP, and at higher levels compared to MR31 iPS cells. For instance, MAG was expressed in more than 80% of the BC1 derived-OPs versus less than 20% in OPs derived from the MR31 line. Similar results were also shown for MOBP, which was expressed in more than 80% of the BC1 derived-OPs versus 30% of MR31-derived OPs. The number of the positively stained cells was divided by the total cell number to estimate the percent of cells that expressed a marker. Each experiment was replicated at least 3 times from 3 independent differentiation experiments. Despite expression of MAG and MOBP proteins in the iPS-OPs, MBP immunostaining was not detected in any of the iPS-OPs lines. This funding corroborates what we have previously shown for human ES-derived OPs, where MBP expression was found exclusively in nonproliferating, terminally-differentiated oligodendrocytes with the addition of T3 to the cell culture media [24].

### OPs Survive and Differentiate after Transplantation

**Fig 5:**
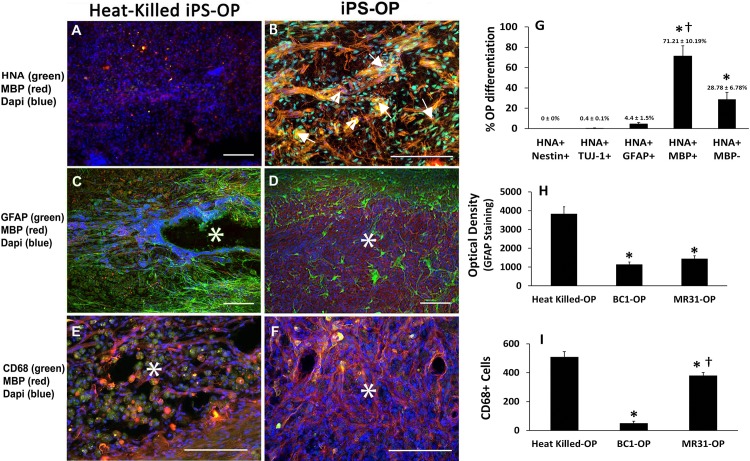
Immunohistological characterization in rat spinal cords at the epicenter of injury (asterisks) following transplantation of either (A) heat-killed iPS-OPs or (B) live iPS-OPs, 24 hours after injury. Human specific anti-nuclei antibody (HNA) staining showed that cells transplanted at the site of injury could survive, differentiate and express myelin basic protein (MBP, red) in iPS-OPs group, compared to the heat-killed iPS-OPs group. (C) Heat-killed iPS-OPs treated spinal cords demonstrated more cavitation and higher glial fibrillary acidic protein (GFAP) (green) expression than (D) spinal cords treated with iPS-OPs. Heat-killed iPS-OPs also expressed less MBP, a mature oligodendrocyte marker, than live iPS-OPs (red). (E) Double immunostaining with CD68 (expressed by macrophages, microglial, and T cells) and MBP shows more CD68+/MBP+ cells in control animals with morphology indicative of active macrophages or microglial engulfment of OPs. (F) Spinal cords injected with iPS-OPs had significantly fewer CD68+/MBP+ cells. Tissues shown here were isolated approximately 2 months after transplantation. DAPI was used to stain rat and human nuclei (blue). Scale bars for A, C, D: 100 μm and B, E, F: 30 μm. (G) Quantitative image analysis shows that more than 70% of HNA+ cells were differentiated MBP+ oligodendrocytes. Only 4.4 ± 1.5% of grafted cells express glial fibrillary acidic protein (GFAP), while no HNA+ cells were detected with co-localized staining for neuronal marker neuron-specific class III beta-tubulin (TUJ1) or NESTIN. Values represent mean ± SEM. * denote statistical significance of human MBP+ oligodendrocytes and MBP- OPs compared to either human-derived neural progenitors (Nestin+), neurons (TUJ1+) or astrocytes (GFAP+) and † denotes statistical significance of human HNA+MBP+ compared to HNA+MBP- OPs by Tukey post hoc tests. Tissue slices encompassing the injury epicenter were stained in intervals for each antibody combination from 4 rats in each treatment group, P < 0.001). (H) Optical density quantification of GFAP staining, which shows significantly higher intensity of GFAP staining in heat-killed iPS-OP compared with iPS-OPs of both BC1 and MR31 lines. (I) Quantification of the number of CD68+ cells shows significantly higher CD68+ cells in heat-killed iPS-OPs, indicative of increased inflammation and macrophage activation, compared with iPS-OPs of both BC1 and MR31 lines. Values in the graphs represent mean ± SEM. * and † denote statistical significance compared to heat-killed iPS-OPs and BC1-OPs, respectively by Tukey post hoc tests (10 identically numbered tissue slices encompassing the injury epicenter from 4 rats were analyzed for each treatment group, P < 0.001).

iPS-derived OPs were transplanted into rat spinal cords 24 hours after a moderate contusive SCI. The BC1 line was used for the *in vivo* assessments because this line expressed the greatest amount of late-OP markers compared to the other lines tested. Immunohistological analysis of spinal cords two months after cell transplantation showed human iPS-OP survival and integration into the spinal cord without tumor formation (Fig. 5). The site of cell injection can be easily identified as the regions of clustered, transplanted cells in areas with damage to the endogenous tissue. Human cells were confirmed by human anti-nuclei antibody (HNA), which is not detected in spinal cords injected with heat-killed iPS-OPs (Fig. 5A). In contrast, patches of cells that co-express HNA (green labeled) and MBP (red labeled) were detected at the injection site encompassing the injury epicenter in spinal cords transplanted with iPS-OPs (Fig. 5B). HNA staining indicates survival of the transplanted iPS-OPs while co-localization of HNA and MBP protein confirms OP differentiation of these cells into mature oligodendrocytes.

Next, we examined the extent of glial scarring by studying the number GFAP+ astrocytes and the level of staining around the site of injury. Results show increased migration of GFAP+ cells to the site of injury and higher intensity of GFAP staining surrounding cavities in heat-killed iPS-OP controls (Fig. 5C) compared to iPS-OP treated spinal cords (Fig. 5D). Immune cells have also been demonstrated in the spinal cords of immunocompromised rats that engulf transplanted cells and show immunostaining for OP markers [49,50]. To investigate this possibility, we dual-stained sections with MBP and CD68, a marker of multiple types of immune cells activated in the damaged spinal cord including microglia, macrophages and T cells. Under normal conditions these cells express basal levels of CD68, which then become elevated when they are activated [51]. Two months after transplantation, results showed higher numbers of CD68+ cells in regions treated with heat-killed iPS-OP controls (Fig. 5E) compared to live iPS-OPs. The presence of CD68+ cells in the spinal cord of control rats after cyclosporine is consistent with others that show that the same concentration of cyclosporine reduces but not eliminate CD68+ related activation in this tissue [52]. In Fig. 5F, expression of MBP was seen in the region of transplantation while only a few CD68 expressing cells could be seen in the same region of the iPS-OP transplants. Thus, it seems that the transplanted human cells expressing MBP were not being attacked by the host immune cells. This result may be due in part to the treatment of cyclosporine used in these rats to inhibit graft rejection. It is interesting that the same mechanism involved in cyclosporine treatment that prevents the rejection of the human cell transplants does not prevent the engulfment of endogenous rat oligodendrocytes, which occurs in the control groups. We observed a much larger number of rounded, foamy-like microglial shaped cells that were CD68+ and MBP+ in the control groups injected with heat-killed cells (Fig. 5E). These results suggest that human iPS-OPs may facilitate specific anti-microglial immune responses in the rat spinal cord after injury.

Quantitation analysis was also performed on a series of dual staining experiments from cryosections of spinal cords. HNA+ staining was compared with a variety of markers to identify the differentiated state of the injected iPS-OP BC1 line (Fig. 5G). Staining was also performed with iPS-OPs derived from the other 2 lines with similar outcomes (data not shown). These results showed that HNA+ cells that co-labeled with MBP antibody comprised 71.2 ± 10.19% of the total number of injected HNA+ cells found in the dorsal column. The remaining HNA- MBP positive cells suggest that endogenous rat OPs also infiltrate the injured region. Some reports have suggested that pluripotent stem cell-derived OPs can differentiate into astrocytes [53]. Therefore, the expression of the astrocyte marker GFAP was also studied in these sections. Cells that co-labeled with human nuclei and an astrocyte marker GFAP comprised 4.4 ± 1.5% of the total number of human nuclei+ cells. The small percentage of GFAP staining in the HNA+ cells affirms the robustness of this protocol for differentiating stem cells into OPs. On the other hand, only 0.4 ± 0.1% of transplanted cells co-expressed HNA and the neuronal marker TUJ-1, and there was no co-expression of HNA and the early neural marker (NESTIN) detected in our transplanted cells. Therefore, the OPs derived in culture were not a more primitive progenitor, which could generate a large number of neurons or astrocytes after transplantation. Importantly, these results show that a sufficient number of human iPS-derived OPs survive at least two months after being transplanted 24 hours post-SCI. These unprecedented results show the valuable potential of OPs for early interventions to treat SCI, which may enhance future outcomes of SCI patients by reducing glial scarring and dampening long-acting or overactive immune responses that are known to hamper recovery.

### OPs Reduce Cavitation after SCI

**Fig 6:**
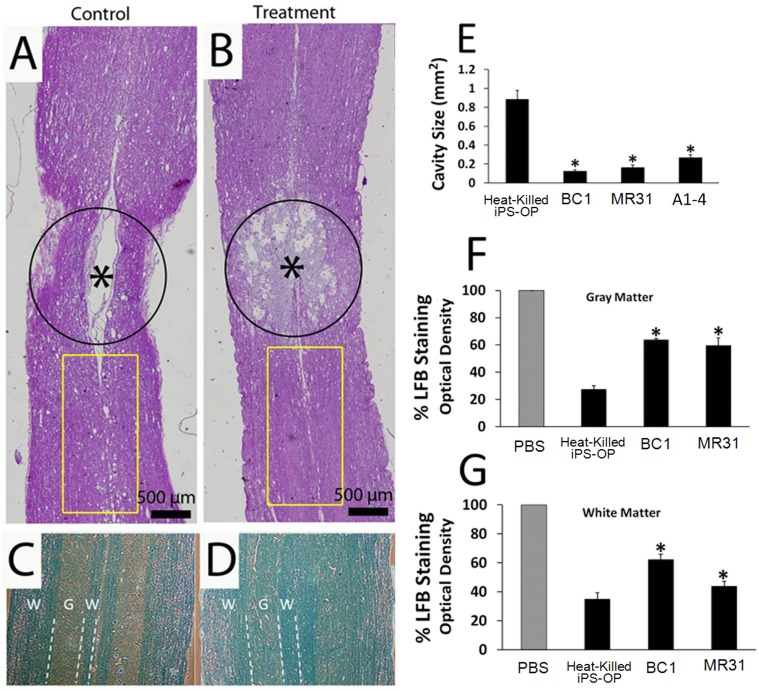
Hematoxylin and eosin and luxol fast blue stained paraffin sections of spinal cords from animals subjected to moderate contusive SCI and treated with (A) Heat-killed iPS-OPs or (B) Live, intact iPS-OPs derived from the BC1 line. As expected for a 12.5 mm contusive injury, cavity formation was seen in both groups; however, the cavitation in spinal cords treated with iPS-OPs did not form well defined glial-like scar structures around the cavity periphery, compared to the control group. Thus, controls showed more pronounced cavitation, which extended further from the epicenter along the central canal. This indicates that the transplantation of live iPS-OPs into the newly formed cavity 24 hours after injury limited the normal expected progression of this level of contusive injury. The asterisks indicate the center of the injury site. Yellow boxes in A and B correspond to adjacent tissue sections stained in C and D, respectively. In these areas immediately surrounding the center of injury, staining with Luxol Fast Blue (LFB) to identify myelin showed reduced staining in the control group (C) versus the iPS-OP transplanted spinal cords (D). Thus, higher levels of LFB in the iPS-OP treated rats may indicate the presence of greater amounts of myelin. The representative spinal cords shown in (A-D) were harvested approximately 2 months after transplantation. Dotted lines help delineate regions of white matter (W) and grey matter (G). (E) Area measurements across groups revealed nearly a 5-fold significant reduction in cavity size in the iPS-OP treated groups compared to the heat-killed controls. Optical density of LBF, normalized to the optical density of the sham control group, was calculated for the grey matter (F) and white matter (G) of spinal sections to quantify the amount of myelin present in each area of the cord. Values in the graphs represent mean ± SEM. * denote statistical significance compared to heat-killed iPS-OPs by Tukey post hoc tests. For each stain, 5 identically numbered tissue slices encompassing the injury epicenter from 4 rats were analyzed for each treatment group, P < 0.001.

To study cavitation and morphological changes at the injury epicenter following cell transplantation, longitudinal sections of injured spinal cords were analyzed by H&E staining (Fig. 6A-B) and electron microscopy. Cavity formation within the gray and white matter surrounding the epicenter of injury are normally observed in untreated spinal cords following a moderate contusive injury. However, two months after implantation, the cavities manifest differently in spinal cords transplanted with control heat-killed iPS-OPs compared to those transplanted with the BC1 iPS-derived OPs. The extent of cavitation was 5-fold larger and spread further from the epicenter in controls (Fig. 6A) compared to iPS-treated spinal cords (Fig. 6B). Longitudinal sections of the cords were also stained with Luxol fast blue (LFB) to analyze the extent of myelination after treatment in the areas surrounding the cavitation (Fig. 6C-D). As expected in all spinal cords, the intensity of LFB staining is greater in the white matter compared to the grey matter due to the presence of myelinated tracts. Spinal cords injected with heat-killed controls (Fig. 6C) showed decreased staining compared to those injected with live BC1 iPS-derived OPs in the white matter tracts (Fig. 6D). This finding suggests remyelination is occurring as a result of the injected cells. In addition, a greater number of cells with dark blue cytoplasmic staining could be seen in the control group compared to the treated group (indicated by arrows, Fig. 6C), which is representative of increased demyelination and macrophage phagocytosis of disintegrated myelin in the control group [54]. This raises the possibility that iPS-OPs may provide immunoprotection to the tissue similar to that observed after MSC and neural stem cell transplantation [55]. Consistent with these findings, the heat-killed control group demonstrated a significantly higher number of cells with dark cytoplasmic LFB staining compared to OPs-treated cords as shown in Fig. 6C-D.

### OPs Remyelinate Axons after SCI

**Fig 7:**
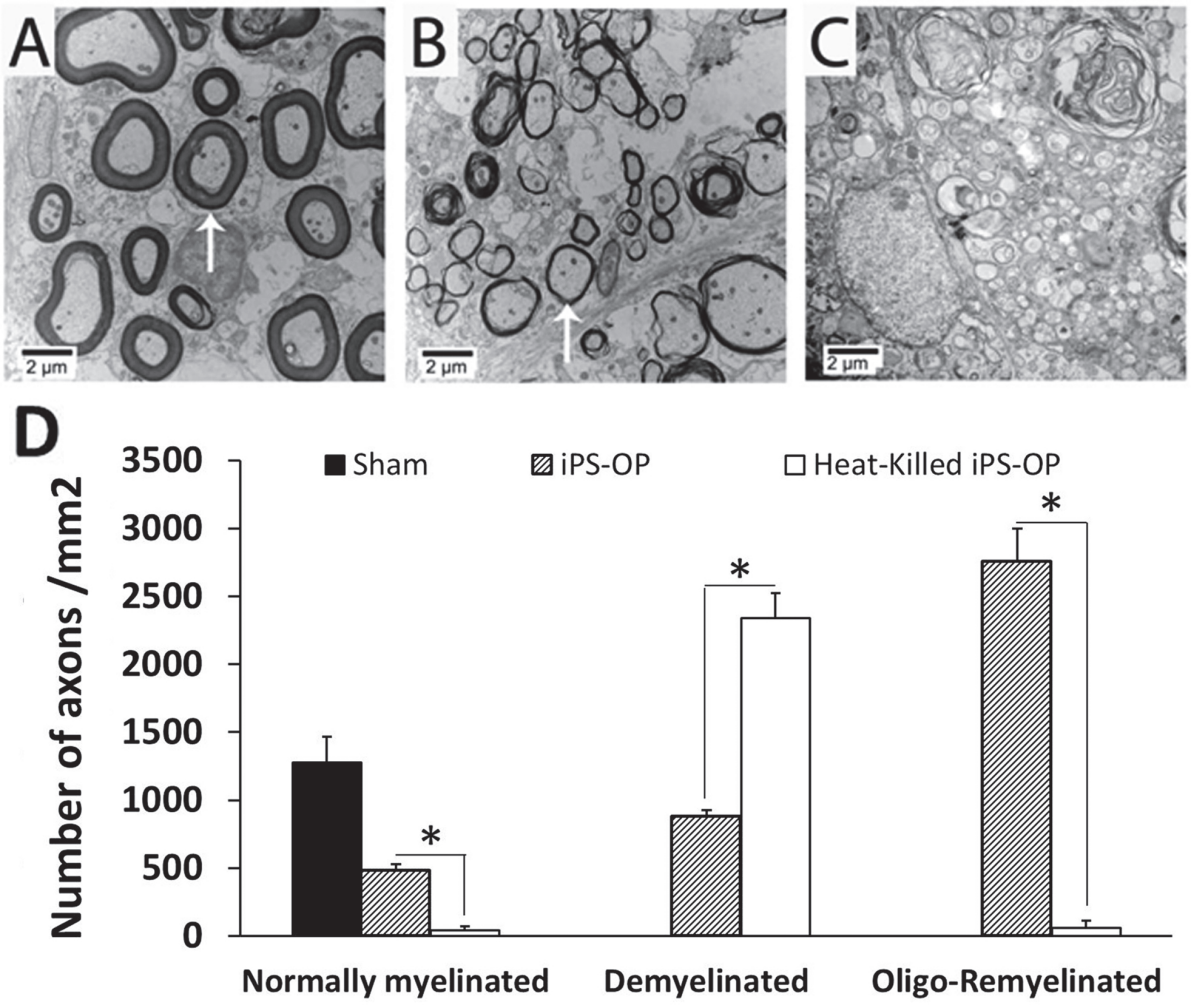
Transmission electron microscopy of transverse sections of the spinal cord showed remyelination of spared axons by BC1-derived iPS-OP cells after contusive SCI in rats. (A) Normal, endogenous myelination of an uninjured rat spinal cord (laminectomy sham control) as indicated by white arrows, showing thick and tightly-packed myelin sheaths. (B) Remyelination observed in rats transplanted with BC1-derived OPs, approximately two months after transplantation. These are sites where oligodendrocytes are detected, as shown in Fig. 4. The relatively thinner and loosely packed myelin sheaths may be compared with the normal condition in (A), suggesting that this is remyelination by human OPs. (C) Multiple nude axons are visible in rats injected with heat-killed iPS-OPs, with no observable remyelination, approximately two months after transplant. (D) Quantification shows that transplantation of BC1-derived OPs resulted in a significant increase in the number of remyelinated axons and reduced demyelinated of axons compared with the heat-killed iPS-OP group. Values in the graphs represent mean ± SEM. * denote statistical significance compared to heat-killed iPS-OPs by Tukey post hoc tests. Ten images encompassing the injury epicenter from 4 rats were analyzed for each treatment group (P < 0.001). Magnification (A-C): 10000X.

Increased levels of myelin were demonstrated in rats treated with iPS-derived OPs using transmission electron microscopy (**TEM**) (Fig. 7A-C). The spinal cord injected with control heat-killed iPS-OPs showed no evidence of myelination with multiple bare axons or severe disruption of myelin sheaths encompassing the injury site. On the other hand, spinal cords injected with BC1-derived iPS-OPs showed evidence of remyelination, where the myelin sheaths are much thinner with loosely arranged lamellae compared to normal myelination detected in an the uninjured spinal cord. Quantification of myelination indicated that the number of remyelinated axons was significantly higher in the iPS-OP transplanted group compared with heat-killed iPS-OP transplanted group (Fig. 7D). iPS-OP transplanted animals also demonstrated significantly less demyelinated axons compared with heat-killed iPS-OP transplanted animals. Notably, heat-killed iPS-OPs-transplanted animals contained an average of 60 oligodendrocyte remyelinated axons per mm^2^ whereas iPS-OP transplanted animals contained an average of 2760 oligodendrocyte remyelinated axons per mm^2^, indicating a 46-fold increase in remyelination in iPS-OP transplanted animals over the heat-killed controls. In addition, more normally myelinated axons were found at the injury epicenter in iPS-OP transplanted spinal cords whereas none were visible in controls.

### Motor Behaviorial Analysis

**Fig 8:**
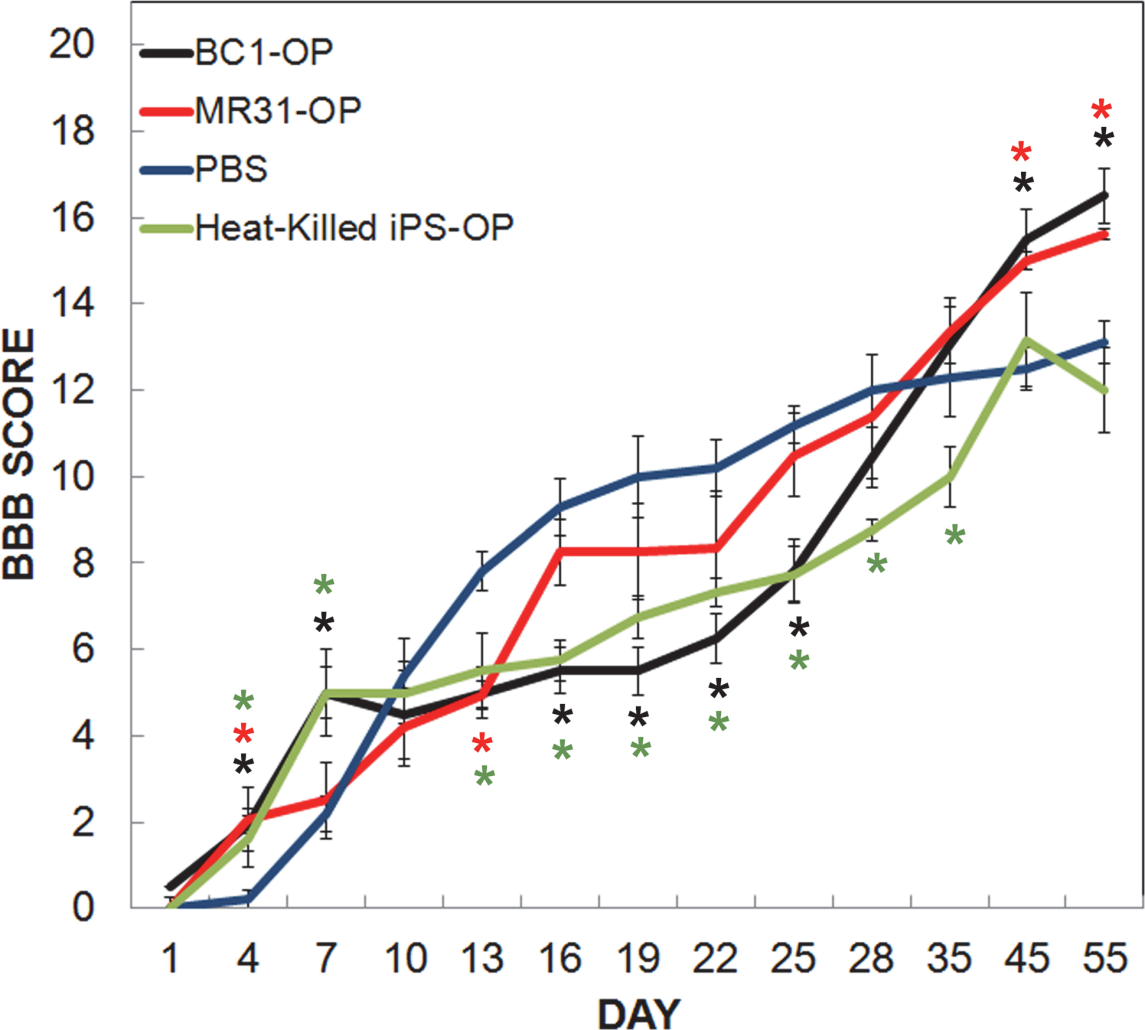
Behavioral assessment 8 weeks following moderate contusive injury (12.5mm) and cell transplantation in rats. The average of the Basso, Beattie, and Bresnahan (BBB) Locomotor Rating Scale (BBB SCORE) for left and right limb movement was evaluated for 55 days following spinal cord injury in rats injected with either PBS (blue), 500,000 heat-killed iPS-OP cells (green), live BC1-OP (black), or MR31-OP (red) cells 24 hrs after injury. Values in the graphs represent mean ± SEM of both left and right limbs as there were no significant differences in response between right and left limb scores. Statistical significance is denoted by a colored asterisk for each cell line with matched color compared to the PBS control group determined by one-way ANOVA followed by a two-way Student’s t-test assuming equal variances (P < 0.025).

BBB analyses demonstrated in interesting trend in BBB scores among groups (Fig. 8). For instance, motor recovery was initially greater after transplantation in rats either injected with live or heat-killed iPS-OPs compared to the control group. However, this effect diminished after the first week and throughout the first month of recovery during which time rats injected with PBS alone showed higher scores. This trend was followed by rats injected with iPS-OPs which showed a significant increase in BBB scores 5 weeks after injection. BBB scores from rats injected with A1-4 derived OPs showed results similar to MR31 (data not shown). Rats injected with heat-killed iPS-OPs were similar to rats injected with PBS at the end of the study.

## Discussion

In this study, we show that OPs can be efficiently derived from human iPS cells from multiple sources using culture methods similar to those we have previously described [24–26]. We generated iPS-OPs from 3 distinct iPS cell lines using 3 different reprogramming strategies. These lines included the BC1 line that was generated with non-integrating, episomally-mediated delivery of the reprogramming factors (*OCT4 (O)*, *SOX2 (S)*, *KLF4 (K)*, *MYC (M)*, *LIN28*) from adult peripheral blood monocytes and 2 other lines generated from bone marrow-derived, mesenchymal stem cells (A1-4 or HMGA1-OSKM) and fetal lung fibroblasts (MR31) by retroviral delivery of either HMGA1-OSKM factors or OSK, respectively [20–22]. The varied reprogramming protocols and origins of cell lines suggest that this approach will be effective in generating OPs of many diverse iPS cell lines.

As demonstrated by our gene expression analyses, iPS-OPs expressed oligodendrocyte-associated markers, including *NG2*, *MOBP*, and *MAG*. Importantly, iPS-OPs lacked the expression of iPS/ES cell signature pluripotent genes (*OCT4*, *NANOG*, or *SOX2)*, indicating that they had differentiated along the neural lineage and were no longer pluripotent. Based on prior work with ES, we distinguished multiple stages of development between EBs and mature oligodendrocytes from these 3 iPS lines. These included neural progenitor (NP), glial progenitor (GP) and OP stages, each of which expresses a specific set of genes and protein markers. Following differentiation to the NP stage, greater than 80% of the cells expressed the early neural markers NESTIN and A2B5. At the GP stage, which is generated by the addition of EGF to the growth media, more than 80% of the cells also expressed NG2 and the PDGFα receptor, in addition to NESTIN and A2B5. Difference between lines became apparent when differentiation to the OP stage was induced by the addition of PDGFα to the culture medium. In OPs derived from the BC1 line, only 30% of cells were positive for the early neural marker NESTIN and more than 60% of cells expressed the OP marker, O1. NESTIN expression in OPs derived from the retroviral-derived iPS lines was nearly double at 60% and O1 expression correspondingly lower than that in BC1 demonstrating their low efficiency at differentiating from their NP and GP stages into OPs. It will be interesting in future studies to determine whether there is a consistent difference in the ability between non-integrative and integrative approaches for deriving iPS to differentiation in various subtypes.

While other studies have look at the differentiation efficiency of iPS lines compared to ES cells [20,21,56], it is not clear whether there are differences in the differentiation efficiency between iPS cells generated by integrating and non-integrating approaches. Interestingly, studies have shown that iPS cell lines vary in their differentiation potentials, and it is possible that vector integration contributes to these differences [57]. No study to date has compared integration-free iPS and nonintegrated iPS derived OPs. Such a study would require a large number of iPS-derived lines and hundreds of experimental animals to validate the results. Although confirmatory studies are needed, we found that the iPS cells derived using episomal vectors differentiate into OPs more efficiently compared to the iPS cells derived using retroviral vectors.

Furthermore, it is unknown whether the variability of different iPS lines are more dependent on the method of reprogramming than their cell of origin. Indeed others have demonstrated that iPS differentiation is highly variable compared to ES cells and dependent in part on their cellular origin. For instance, different lines have demonstrated varying capacities to differentiate into unique cell types [57], and evidence suggests that this is a result of genetic and epigenetic differences between the iPS cell lines and their clones [58,59]. Based on these studies, iPS that are derived from cells of a younger developmental time period may be more “stem-like”, and therefore may respond to differentiation more efficiently. However, we showed that BC1 derived from adult blood generated more cells that expressed late-OP markers compared to both MR31 and A1-4, which are derived from earlier developmental tissue sources (fetal fibroblasts and early blood progenitors, respectively). Likewise, MR31 and A1-4 expressed more immature markers of glial and neural progenitors. These data may suggest that the differentiating capacity of iPS cells is also influenced by the method of iPS generation. Thus, this finding demonstrates a new proof-of-principle that given an effective differentiating environment, iPS cells can differentiate into early glial progenitors with relatively high efficiency regardless of their mode of generation and original cell of origin. In addition, it emphasizes the importance of developing effective protocols in eliciting the efficiency of IPS to differentiation intolate stage OPs for transplantation.

One issue that raises concerns regarding OP transplants is the role of the immune response at the injured area, such as at the site of spinal cord contusion. It is well known that under normal conditions during which animals are not immunosuppressed, macrophage infiltration and glial scarring is increased after SCI [60–62]. This in part is due to the role of astrogliosis, which contributes to the secondary injury phase of SCI. Reactive astrocytes and microglia are major contributors of this process. In response to SCI, astrocytes undergo astrogliosis during which they become proliferative, hypertrophic and exhibit molecular changes that induce scar and cavity formation. These reactive astrocytes appear immediately after injury, and scar formation appears at 3 days [61,62]. By day 3, the initial effects of astrogliosis and scar formation are beneficial for containing the primary injury. However, by day 7 in the rat, the expansion of the glial scar and hyperactivity of reactive astrocytes ultimately leads to progressive damage of the spinal cord. Several studies have indicated that the failure of remyelination is correlated with the presence of this astroglial scar [63–65]. Keirstead et al. demonstrated that progressive astrogliosis after SCI is correlated with the failure of myelinogenic transplants to successfully remyelinate axons if the cells are transplanted after secondary injury has commenced [66]. Our study showed increased migration of GFAP+ cells to the site of injury and higher intensity of GFAP staining surrounding cavities in heat-killed iPS-OP controls compared to live iPS-OP treated animals. This suggests that iPS-OP transplantation 24 h after SCI can attenuate astrogliosis and reduce inflammation. Interestingly, this finding is consistent with other reports showing reduced inflammation and astrogliosis following MSC transplantation following SCI [7]. MSCs as well as other stem cell therapies that reduce inflammation may also suggest that the increases in motor behavioral recovery seen in rats with a single injection of heat-killed iPS-OPsheat-killed iPS-OPs are due to secreted factors from these cells [67]. Despite these promising results after injection, this effect from heat-killed OPs did not facilitate motor behavior or anatomical recovery in the weeks that followed transplantation. It is possible, however, that multiple injections during this time period may warrant better outcomes.

Moderate contusive SCIs typically result in tissue loss at the epicenter of injury, producing a cavity that is essentially devoid of living cells. Cavitation is in part a result of activated astrocytes, or reactive astrocytes, and glial macrophages, known as microglia, that are responsible for the early formation of the glial scar that surrounds the cavity. Glial scarring is important to prevent further bleeding and destruction to the surrounding spinal cord. However, after activation, glial cells continue to increase scar formation such that it impedes oligodendrocyte and axonal regeneration through and around the cavity, impeding recovery [68]. Despite formation of these cavities, which can consume much of the dorsal area of the cord, the majority of contusive injuries also contain some degree of spared parenchyma across the site of lesion, dependent on the injury severity. These areas consist of neuro-pathways that are anatomically intact but electrically defunct due to the destruction of oligodendrocytes and consequent axonal demyelination. Furthermore, we found that transplantation of live iPS-OP into rat spinal cords led to a 5-fold reduction in cavity size compared with animals that received heat-killed cells. Specifically, histological characterization showed neurons and the presence of myelin at the injured site where the OPs were transplanted. This finding indicates that the transplantation of live iPS-OPs into the area where the normal progression of cavity formation occurs, or 24 h after injury, limited the normal expected progression of the cavity formation. Most importantly, reducing the extent of the glial scar and cavity size is paramount for allowing neural regeneration and repair through the site of injury and recovery from SCI. Therefore, iPS-OPs remain a viable option to pursue for future therapy.

Immunohistochemical analyses provided additional insight into the behavior of the iPS-derived cells when transplanted 24 h after contusive SCI. Staining with human specific antibodies showed that the injected iPS-OPs survived in the rat spinal cord and could be detected for the duration of the study, up to two months after transplantation. The cells also efficiently differentiated into oligodendrocytes and distributed over short distances of the spinal cord. H&E-stained sections of the injury epicenter and injection site revealed the presence of cells or tracts contiguous with the parenchyma around the injury epicenter. LFB staining showed a greater presence of myelin encompassing and immediately surrounding the injury epicenter in rats treated with iPS-derived OPs compared with heat-killed controls. This could be due to grafted OPs re-myelinating the axonal tracts proximal to injury, although further studies are needed to establish this conclusively, as LFB staining also detected endogenous spared myelin. In addition, only a small percentage of transplanted cells expressed antigenic markers of astrocytes or other neural lineages. This finding, together with the *in vitro* immunocytochemical profiling of the cell populations, indicate effective differentiation of cells to mature oligodendrocytes and a lack of reversion to a more pluripotent phenotype after transplantation to the injury site. Motor behavorial assestments were also consistent with the histological data at this time which showed that rats injected with iPS-OP cells had great motor recovery than rats injected with either PBS or heat-killed iPS-OPs. It is interesting that an increase in recovery compared to controls was noted approximately one month after OPs were injected into the spinal cord, suggesting that this may be the required time frame for their maturation and sufficient remyelination to detect the earliest benefits of this therapy. What was particularly interesting was the bimodal distribution in BBB scores or motor improvement from iPS-OPs compared to controls (the first week and last weeks showing increased scores in the iPS-OP groups) suggesting that different mechanisms may be contributing to iPS-OP induced recovery. Importantly, the loss of pluripotency underscores that the transplanted cells did not develop teratomas or cysts *in vivo*. Such characteristics are crucial for safe and effective administration of cells as well as tissue repair. These results also lend support to further studies with longer durations to determine the iPS-OPs efficacy for transplantation.

Remyelination by the implanted cells was confirmed using transmission electron microscopy, which showed significantly more myelinated axons in the iPS-OP treated group compared to the heat-killed iPS-OP control group. Transplantation of iPS-derived OPs 24 h after SCI resulted in a 36-fold increase in oligodendrocyte remyelination of axons throughout the white matter in the dorsal column after two months, compared with heat-killed iPS-OPs remyelination in controls. Ensheathment of axons by processes of HNA+-transplanted cells confirmed that at least some remyelination was performed by transplanted OPs. Several studies have suggested that the difference in myelination seen between stem cell injections and control groups indicate remyelination from the injected cells [69]. However, other studies, especially those utilizing MSC transplants, have shown that these myelin fraps may indicate remyelination produced by the rats’ endogenous cells, through trophic factors secreted by iPS-OP transplants that promote endogenous oligodendrocyte survival [70]. Furthermore, some have shown that various glial progenitors derived from human and rodents are also able to myelinate after transplantation [66,71,72]. Thus, we cannot rule out the possibility that our cells may also actively secrete factors that promote myelination generated by the endogenous tissue. However, based on the co-localization of the human specific marker express HNA in the majority of the cells that also express an essential myelin component and marker of mature OLs, these data strongly suggest that human iPS-OPs are contributing to the increased levels of myelin detected by immunostaining, LFB expression, and electron microscopy. Nonetheless, the present study is the first to show that OPs can also be derived from integration-free human iPS cells and that these cells at least partially remyelinate injured axons following SCI.

Previous studies have reported the derivation of OPs from murine iPS [73–75] and from human virally integrated iPS [76,77]. Tokumoto *et al*. [74]utilized iPS generated from fetal mouse fibroblasts using retroviruses and showed O4^+^ early oligodendrocyte progenitors *in vitro*, and Czepiel *et al*. [73] demonstrated myelination from similar cells in the demyelinated corpus callosum of *Shriver-/-* mice. A recent study also reported the *in vitro* differentiation of oligodendrocytes from retrovirally-derived human iPS cells generated from X-linked adrenoleukodystrophy patients [76]. Another study reported the differentiation of retrovirally-derived human iPS cells to OPs and their use in a rat model of optic chiasm demyelination [77]. While these cells provide important models for studying oligodendrocyte development and disease, they cannot be rendered safe for clinical purposes because of the retroviral reprogramming method used to derive them. To date, no study has shown the survival of a non-integrated iPS line to generate OPs for treatment of SCI in a rodent model.

Another concern for the clinical use of iPS cells from a recent report in mice that showed iPS elicit T-cell responses similar that of embryonic stem cells [78]. Interestingly, this same study showed that iPS generated by non-integrative methods produced significantly lower responses compared to iPS generated by virus when injected in the same isogenic strain of mice. Although our study utilized immune-tolerant rats and thus cannot address the mechanisms involved in this type of response, our results do show that BC1 iPS-OPs survive, integrate, and facilitate myelination within the spinal cord parenchyma in a fashion similar to those generated with virus. Thus, BC1-derived OPs can provide a suitable source of OPs that would potentially eliminate this issue in clinical trials.

In conclusion, we show that early transplantation of human iPS derived OPs reduced cavitation and glial scarring up to two months after transplantation. Heat-killed iPS-OP controls and co-localization of the human specific marker and the mature oligodendrocyte marker MBP suggest that these effects are dependent on live iPS-OP survival and differentiation at the epicenter of injury. Moreover, OP differentiation from human stem cell transplantation has been less efficient compared to mouse models, and we show that their efficiency *in vivo* is dependent in part on the media and growth factors employed in its cell culture. For this purpose, we show the ability to derive in high purity oligodendrocyte progenitors from human iPS lines derived by a variety of sources including an episomal vector.

## Supporting Information

S1 Checklist ARRIVE Guidelines for Research Involving Spinal Cord Injury. (DOCX)

S1 Table The source of the iPS cell lines and the reprogramming method. Three different iPS cell lines were used in this study: BC1, MR31 and A1-4. The sources of the these cells and the reprogramming methods used are summarized in this table.
(DOCX)

S2 Table List of the primary antibodies. (DOCX)

S3 Table List of genes tested for real time-PCR. (DOCX)

## References

[1] Gunaseeli I, Doss MX, Antzelevitch C, Hescheler J, Sachinidis A (2010) Induced pluripotent stem cells as a model for accelerated patient- and disease-specific drug discovery. Curr Med Chem 17: 759–766. 20088756 10.2174/092986710790514480 PMC2844480

[2] Lau F, Ahfeldt T, Osafune K, Akustsu H, Cowan CA (2009) Induced pluripotent stem (iPS) cells: an up-to-the-minute review. F1000 Biol Rep 1: 84 10.3410/B1-84 20948605 PMC2948253

[3] Nishikawa S, Goldstein RA, Nierras CR (2008) The promise of human induced pluripotent stem cells for research and therapy. Nat Rev Mol Cell Biol 9: 725–729. 10.1038/nrm2466 18698329

[4] Wang S, Bates J, Li X, Schanz S, Chandler-Militello D, et al (2013) Human iPSC-derived oligodendrocyte progenitor cells can myelinate and rescue a mouse model of congenital hypomyelination. Cell Stem Cell 12: 252–264. 10.1016/j.stem.2012.12.002 23395447 PMC3700553

[5] Yoshihara H, Shumsky JS, Neuhuber B, Otsuka T, Fischer I, et al (2006) Combining motor training with transplantation of rat bone marrow stromal cells does not improve repair or recovery in rats with thoracic contusion injuries. Brain Research 1119: 65–75. 17027672 10.1016/j.brainres.2006.08.080

[6] Torres-Espin A, Redondo-Castro E, Hernandez J, Navarro X (2014) Bone marrow mesenchymal stromal cells and olfactory ensheathing cells transplantation after spinal cord injury—a morphological and functional comparison in rats. Eur J Neurosci 39: 1704–1717. 10.1111/ejn.12542 24635194

[7] Kumagai G, Tsoulfas P, Toh S, McNiece I, Bramlett HM, et al (2013) Genetically modified mesenchymal stem cells (MSCs) promote axonal regeneration and prevent hypersensitivity after spinal cord injury. Exp Neurol 248: 369–380. 10.1016/j.expneurol.2013.06.028 23856436

[8] Wang LJ, Zhang RP, Li JD (2014) Transplantation of neurotrophin-3-expressing bone mesenchymal stem cells improves recovery in a rat model of spinal cord injury. Acta Neurochir (Wien) 156: 1409–1418. 10.1007/s00701-014-2089-6 24744011

[9] Mohammad-Gharibani P, Tiraihi T, Delshad A, Arabkheradmand J, Taheri T (2013) Improvement of contusive spinal cord injury in rats by co-transplantation of gamma-aminobutyric acid-ergic cells and bone marrow stromal cells. Cytotherapy 15: 1073–1085. 10.1016/j.jcyt.2013.05.002 23806239

[10] Penha EM, Meira CS, Guimaraes ET, Mendonca MV, Gravely FA, et al (2014) Use of autologous mesenchymal stem cells derived from bone marrow for the treatment of naturally injured spinal cord in dogs. Stem Cells Int 2014: 437–445. 10.1155/2014/437521 PMC3956412 24723956

[11] Reeves A, Keirstead HS (2012) Stem cell based strategies for spinal cord injury repair. Adv Exp Med Biol 760: 16–24. 23281511 10.1007/978-1-4614-4090-1_2

[12] Quertainmont R, Cantinieaux D, Botman O, Sid S, Schoenen J, et al (2012) Mesenchymal stem cell graft improves recovery after spinal cord injury in adult rats through neurotrophic and pro-angiogenic actions. PLoS One 7: e39500 10.1371/journal.pone.0039500 22745769 PMC3380009

[13] Lu P, Kadoya K, Tuszynski MH (2014) Axonal growth and connectivity from neural stem cell grafts in models of spinal cord injury. Curr Opin Neurobiol 27: 103–109. 10.1016/j.conb.2014.03.010 24709371

[14] Chang YW, Goff LA, Li H, Kane-Goldsmith N, Tzatzalos E, et al (2009) Rapid induction of genes associated with tissue protection and neural development in contused adult spinal cord after radial glial cell transplantation. J Neurotrauma 26: 979–993. 10.1089/neu.2008-0762 19257808 PMC2848950

[15] Ourednik J, Ourednik V, Lynch WP, Schachner M, Snyder EY (2002) Neural stem cells display an inherent mechanism for rescuing dysfunctional neurons. Nat Biotechnol 20: 1103–1110. 12379867 10.1038/nbt750

[16] Teng YD, Lavik EB, Qu X, Park KI, Ourednik J, et al (2002) Functional recovery following traumatic spinal cord injury mediated by a unique polymer scaffold seeded with neural stem cells. Proc Natl Acad Sci U S A 99: 3024–3029. 11867737 10.1073/pnas.052678899 PMC122466

[17] Kerr DA, Llado J, Shamblott MJ, Maragakis NJ, Irani DN, et al (2003) Human embryonic germ cell derivatives facilitate motor recovery of rats with diffuse motor neuron injury. J Neurosci 23: 5131–5140. 12832537 10.1523/JNEUROSCI.23-12-05131.2003 PMC6741166

[18] Park DY, Mayle RE, Smith RL, Corcoran-Schwartz I, Kharazi AI, et al (2013) Combined Transplantation of Human Neuronal and Mesenchymal Stem Cells following Spinal Cord Injury. Global Spine J 3: 1–6. 10.1055/s-0033-1337118 24436845 PMC3854610

[19] All AH, Bazley FA, Gupta S, Pashai N, Hu C, et al (2012) Human embryonic stem cell-derived oligodendrocyte progenitors aid in functional recovery of sensory pathways following contusive spinal cord injury. PLoS One 7: e47645 10.1371/journal.pone.0047645 23091637 PMC3473046

[20] Shah SN, Kerr C, Cope L, Zambidis E, Liu C, et al (2012) HMGA1 reprograms somatic cells into pluripotent stem cells by inducing stem cell transcriptional networks. PLoS One 7: e48533 10.1371/journal.pone.0048533 23166588 PMC3499526

[21] Ye Z, Zhan H, Mali P, Dowey S, Williams DM, et al (2009) Human-induced pluripotent stem cells from blood cells of healthy donors and patients with acquired blood disorders. Blood 114: 5473–5480. 10.1182/blood-2009-04-217406 19797525 PMC2798863

[22] Chou BK, Mali P, Huang X, Ye Z, Dowey SN, et al (2011) Efficient human iPS cell derivation by a non-integrating plasmid from blood cells with unique epigenetic and gene expression signatures. Cell Res 21: 518–529. 10.1038/cr.2011.12 21243013 PMC3193421

[23] Cheng L, Hansen NF, Zhao L, Du Y, Zou C, et al (2012) Low incidence of DNA sequence variation in human induced pluripotent stem cells generated by nonintegrating plasmid expression. Cell Stem Cell 10: 337–344. 10.1016/j.stem.2012.01.005 22385660 PMC3298448

[24] Kerr CL, Letzen BS, Hill CM, Agrawal G, Thakor NV, et al (2010) Efficient differentiation of human embryonic stem cells into oligodendrocyte progenitors for application in a rat contusion model of spinal cord injury. Int J Neurosci 120: 305–313. 10.3109/00207450903585290 20374080

[25] Letzen BS, Liu C, Thakor NV, Gearhart JD, All AH, et al (2010) MicroRNA expression profiling of oligodendrocyte differentiation from human embryonic stem cells. PLoS One 5: e10480 10.1371/journal.pone.0010480 20463920 PMC2864763

[26] Chaerkady R, Kerr CL, Marimuthu A, Kelkar DS, Kashyap MK, et al (2009) Temporal analysis of neural differentiation using quantitative proteomics. J Proteome Res 8: 1315–1326. 10.1021/pr8006667 19173612 PMC2693473

[27] Chaerkady R, Letzen B, Renuse S, Sahasrabuddhe NA, Kumar P, et al (2011) Quantitative temporal proteomic analysis of human embryonic stem cell differentiation into oligodendrocyte progenitor cells. Proteomics 11: 4007–4020. 10.1002/pmic.201100107 21770034 PMC3728712

[28] Coetzee T, Fujita N, Dupree J, Shi R, Blight A, et al (1996) Myelination in the absence of galactocerebroside and sulfatide: normal structure with abnormal function and regional instability. Cell 86: 209–219. 8706126 10.1016/s0092-8674(00)80093-8

[29] Jakovcevski I, Filipovic R, Mo Z, Rakic S, Zecevic N (2009) Oligodendrocyte development and the onset of myelination in the human fetal brain. Front Neuroanat 3: 1–15. 10.3389/neuro.05.001.2009 19521542 PMC2694674

[30] Onifer SM, Rabchevsky AG, Scheff SW (2007) Rat models of traumatic spinal cord injury to assess motor recovery. ILAR J 48: 385–395. 17712224 10.1093/ilar.48.4.385

[31] Metz GA, Curt A, van de Meent H, Klusman I, Schwab ME, et al (2000) Validation of the weight-drop contusion model in rats: a comparative study of human spinal cord injury. J Neurotrauma 17: 1–17. 10674754 10.1089/neu.2000.17.1

[32] Young W (2002) Spinal cord contusion models. Prog Brain Res 137: 231–255. 12440371 10.1016/s0079-6123(02)37019-5

[33] Agrawal G, Kerr C, Thakor NV, All AH (2010) Characterization of graded multicenter animal spinal cord injury study contusion spinal cord injury using somatosensory-evoked potentials. Spine (Phila Pa 1976) 35: 1122–1127. 20354478 10.1097/BRS.0b013e3181be5fa7 PMC2871968

[34] Bazley FA, Hu C, Maybhate A, Pourmorteza A, Pashai N, et al (2012) Electrophysiological evaluation of sensory and motor pathways after incomplete unilateral spinal cord contusion. J Neurosurg Spine 16: 414–423. 10.3171/2012.1.SPINE11684 22303873

[35] Bazley FA, Maybhate A, Tan CS, Thakor NV, Kerr C, et al (2014) Enhancement of bilateral cortical somatosensory evoked potentials to intact forelimb stimulation following thoracic contusion spinal cord injury in rats. IEEE Trans Neural Syst Rehabil Eng 22: 953–964. 10.1109/TNSRE.2014.2319313 24801738

[36] Bazley FA, Pashai N, Kerr CL, All AH (2014) The effects of local and general hypothermia on temperature profiles of the central nervous system following spinal cord injury in rats. Ther Hypothermia Temp Manag 4: 115–124. 10.1089/ther.2014.0002 25019643 PMC4151073

[37] Maybhate A, Hu C, Bazley FA, Yu Q, Thakor NV, et al (2012) Potential long-term benefits of acute hypothermia after spinal cord injury: assessments with somatosensory-evoked potentials. Crit Care Med 40: 573–579. 10.1097/CCM.0b013e318232d97e 22001581 PMC3261348

[38] Basso DM, Beattie MS, Bresnahan JC (1995) A sensitive and reliable locomotor rating scale for open field testing in rats. J Neurotrauma 12: 1–21. 7783230 10.1089/neu.1995.12.1

[39] Basso DM, Beattie MS, Bresnahan JC (1996) Graded histological and locomotor outcomes after spinal cord contusion using the NYU weight-drop device versus transection. Exp Neurol 139: 244–256. 8654527 10.1006/exnr.1996.0098

[40] Hameed A, Hruban RH, Gage W, Pettis G, Fox WM 3rd (1994) Immunohistochemical expression of CD68 antigen in human peripheral blood T cells. Hum Pathol 25: 872–876. 8088761 10.1016/0046-8177(94)90005-1

[41] Tysseling-Mattiace VM, Sahni V, Niece KL, Birch D, Czeisler C, et al (2008) Self-assembling nanofibers inhibit glial scar formation and promote axon elongation after spinal cord injury. J Neurosci 28: 3814–3823. 10.1523/JNEUROSCI.0143-08.2008 18385339 PMC2752951

[42] Blight AR (1983) Cellular morphology of chronic spinal cord injury in the cat: analysis of myelinated axons by line-sampling. Neuroscience 10: 521–543. 6633870 10.1016/0306-4522(83)90150-1

[43] Durongphongtorn S, McDonell WN, Kerr CL, Neto FJ, Mirakhur KK (2006) Comparison of hemodynamic, clinicopathologic, and gastrointestinal motility effects and recovery characteristics of anesthesia with isoflurane and halothane in horses undergoing arthroscopic surgery. Am J Vet Res 67: 32–42. 16426209 10.2460/ajvr.67.1.32

[44] Graham V, Khudyakov J, Ellis P, Pevny L (2003) SOX2 functions to maintain neural progenitor identity. Neuron 39: 749–765. 12948443 10.1016/s0896-6273(03)00497-5

[45] Dawson MR, Levine JM, Reynolds R (2000) NG2-expressing cells in the central nervous system: are they oligodendroglial progenitors? J Neurosci Res 61: 471–479. 10956416 10.1002/1097-4547(20000901)61:5<471::AID-JNR1>3.0.CO;2-N

[46] Montague P, McCallion AS, Davies RW, Griffiths IR (2006) Myelin-associated oligodendrocytic basic protein: a family of abundant CNS myelin proteins in search of a function. Dev Neurosci 28: 479–487. 17028425 10.1159/000095110

[47] Trapp BD, Andrews SB, Cootauco C, Quarles R (1989) The myelin-associated glycoprotein is enriched in multivesicular bodies and periaxonal membranes of actively myelinating oligodendrocytes. J Cell Biol 109: 2417–2426. 2478568 10.1083/jcb.109.5.2417 PMC2115868

[48] Watanabe M, Sakurai Y, Ichinose T, Aikawa Y, Kotani M, et al (2006) Monoclonal antibody Rip specifically recognizes 2',3'-cyclic nucleotide 3'-phosphodiesterase in oligodendrocytes. J Neurosci Res 84: 525–533. 16786579 10.1002/jnr.20950

[49] Parr AM, Kulbatski I, Tator CH (2007) Transplantation of adult rat spinal cord stem/progenitor cells for spinal cord injury. J Neurotrauma 24: 835–845. 17518538 10.1089/neu.2006.3771

[50] Pawelczyk E, Jordan EK, Balakumaran A, Chaudhry A, Gormley N, et al (2009) In vivo transfer of intracellular labels from locally implanted bone marrow stromal cells to resident tissue macrophages. PLoS One 4: e6712 10.1371/journal.pone.0006712 19696933 PMC2726631

[51] Fleming JC, Norenberg MD, Ramsay DA, Dekaban GA, Marcillo AE, et al (2006) The cellular inflammatory response in human spinal cords after injury. Brain 129: 3249–3269. 17071951 10.1093/brain/awl296

[52] Lu HZ, Wang YX, Zhou JS, Wang FC, Hu JG (2010) Cyclosporin A increases recovery after spinal cord injury but does not improve myelination by oligodendrocyte progenitor cell transplantation. BMC Neurosci 11: 127 10.1186/1471-2202-11-127 20937147 PMC2959094

[53] Erceg S, Ronaghi M, Oria M, Rosello MG, Arago MA, et al (2010) Transplanted oligodendrocytes and motoneuron progenitors generated from human embryonic stem cells promote locomotor recovery after spinal cord transection. Stem Cells 28: 1541–1549. 10.1002/stem.489 20665739 PMC2996083

[54] George R, Griffin JW (1994) Delayed macrophage responses and myelin clearance during Wallerian degeneration in the central nervous system: the dorsal radiculotomy model. Exp Neurol 129: 225–236. 7957737 10.1006/exnr.1994.1164

[55] English K, Mahon BP (2011) Allogeneic mesenchymal stem cells: agents of immune modulation. J Cell Biochem 112: 1963–1968. 10.1002/jcb.23119 21445861

[56] Bilic J, Izpisua Belmonte JC (2012) Concise review: Induced pluripotent stem cells versus embryonic stem cells: close enough or yet too far apart? Stem Cells 30: 33–41. 10.1002/stem.700 22213481

[57] Hu BY, Weick JP, Yu J, Ma LX, Zhang XQ, et al (2010) Neural differentiation of human induced pluripotent stem cells follows developmental principles but with variable potency. Proc Natl Acad Sci U S A 107: 4335–4340. 10.1073/pnas.0910012107 20160098 PMC2840097

[58] Doi A, Park IH, Wen B, Murakami P, Aryee MJ, et al (2009) Differential methylation of tissue- and cancer-specific CpG island shores distinguishes human induced pluripotent stem cells, embryonic stem cells and fibroblasts. Nat Genet 41: 1350–1353. 10.1038/ng.471 19881528 PMC2958040

[59] Gore A, Li Z, Fung HL, Young JE, Agarwal S, et al (2011) Somatic coding mutations in human induced pluripotent stem cells. Nature 471: 63–67. 10.1038/nature09805 21368825 PMC3074107

[60] Ronaghi M, Erceg S, Moreno-Manzano V, Stojkovic M (2010) Challenges of stem cell therapy for spinal cord injury: human embryonic stem cells, endogenous neural stem cells, or induced pluripotent stem cells? Stem Cells 28: 93–99. 10.1002/stem.253 19904738

[61] Antonic A, Sena ES, Lees JS, Wills TE, Skeers P, et al (2013) Stem cell transplantation in traumatic spinal cord injury: a systematic review and meta-analysis of animal studies. PLoS Biol 11: e1001738 10.1371/journal.pbio.1001738 24358022 PMC3866091

[62] Li J, Lepski G (2013) Cell transplantation for spinal cord injury: a systematic review. Biomed Res Int 2013: 786475 10.1155/2013/786475 23484157 PMC3581246

[63] Krsulovic J, Couve E, Roncagliolo M (1999) Dysmyelination, demyelination and reactive astrogliosis in the optic nerve of the taiep rat. Biol Res 32: 253–262. 10983245 10.4067/s0716-97601999000400005

[64] Lucchinetti CF, Bruck W, Rodriguez M, Lassmann H (1996) Distinct patterns of multiple sclerosis pathology indicates heterogeneity on pathogenesis. Brain Pathol 6: 259–274. 8864283 10.1111/j.1750-3639.1996.tb00854.x PMC7161824

[65] Wu E, Raine CS (1992) Multiple sclerosis. Interactions between oligodendrocytes and hypertrophic astrocytes and their occurrence in other, nondemyelinating conditions. Lab Invest 67: 88–99. 1625450

[66] Keirstead HS, Nistor G, Bernal G, Totoiu M, Cloutier F, et al (2005) Human embryonic stem cell-derived oligodendrocyte progenitor cell transplants remyelinate and restore locomotion after spinal cord injury. J Neurosci 25: 4694–4705. 15888645 10.1523/JNEUROSCI.0311-05.2005 PMC6724772

[67] Urdzikova LM, Ruzicka J, LaBagnara M, Karova K, Kubinova S, et al (2014) Human mesenchymal stem cells modulate inflammatory cytokines after spinal cord injury in rat. Int J Mol Sci 15: 11275–11293. 10.3390/ijms150711275 24968269 PMC4139782

[68] Yuan YM, He C (2013) The glial scar in spinal cord injury and repair. Neurosci Bull 29: 421–435. 10.1007/s12264-013-1358-3 23861090 PMC5561940

[69] Nistor GI, Totoiu MO, Haque N, Carpenter MK, Keirstead HS (2005) Human embryonic stem cells differentiate into oligodendrocytes in high purity and myelinate after spinal cord transplantation. Glia 49: 385–396. 15538751 10.1002/glia.20127

[70] Dasari VR, Spomar DG, Gondi CS, Sloffer CA, Saving KL, et al (2007) Axonal remyelination by cord blood stem cells after spinal cord injury. J Neurotrauma 24: 391–410. 17376002 10.1089/neu.2006.0142 PMC1859845

[71] Brustle O, Jones KN, Learish RD, Karram K, Choudhary K, et al (1999) Embryonic stem cell-derived glial precursors: a source of myelinating transplants. Science 285: 754–756. 10427001 10.1126/science.285.5428.754

[72] Keirstead HS, Ben-Hur T, Rogister B, O'Leary MT, Dubois-Dalcq M, et al (1999) Polysialylated neural cell adhesion molecule-positive CNS precursors generate both oligodendrocytes and Schwann cells to remyelinate the CNS after transplantation. J Neurosci 19: 7529–7536. 10460259 10.1523/JNEUROSCI.19-17-07529.1999 PMC6782511

[73] Czepiel M, Balasubramaniyan V, Schaafsma W, Stancic M, Mikkers H, et al (2011) Differentiation of induced pluripotent stem cells into functional oligodendrocytes. Glia 59: 882–892. 10.1002/glia.21159 21438010

[74] Tokumoto Y, Ogawa S, Nagamune T, Miyake J (2010) Comparison of efficiency of terminal differentiation of oligodendrocytes from induced pluripotent stem cells versus embryonic stem cells in vitro. J Biosci Bioeng 109: 622–628. 10.1016/j.jbiosc.2009.11.013 20471604

[75] Tsuji O, Miura K, Okada Y, Fujiyoshi K, Mukaino M, et al (2010) Therapeutic potential of appropriately evaluated safe-induced pluripotent stem cells for spinal cord injury. Proc Natl Acad Sci U S A 107: 12704–12709. 10.1073/pnas.0910106107 20615974 PMC2906548

[76] Jang J, Kang HC, Kim HS, Kim JY, Huh YJ, et al (2011) Induced pluripotent stem cell models from X-linked adrenoleukodystrophy patients. Ann Neurol 70: 402–409. 10.1002/ana.22486 21721033

[77] Pouya A, Satarian L, Kiani S, Javan M, Baharvand H (2011) Human induced pluripotent stem cells differentiation into oligodendrocyte progenitors and transplantation in a rat model of optic chiasm demyelination. PLoS One 6: e27925 10.1371/journal.pone.0027925 22125639 PMC3220701

[78] Zhao T, Zhang ZN, Rong Z, Xu Y (2011) Immunogenicity of induced pluripotent stem cells. Nature 474: 212–215. 10.1038/nature10135 21572395

