# Supplementary material for: Early Intervention for Spinal Cord Injury with Human Induced Pluripotent Stem Cells Oligodendrocyte Progenitors
Source: PLoS One. 2015 Jan 30;10(1):e0116933. doi: 10.1371/journal.pone.0116933 (PMC4311989; doi:10.1371/journal.pone.0116933)

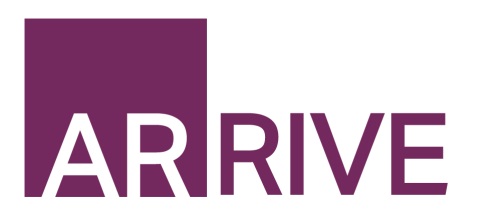


The ARRIVE Guidelines Checklist

Animal Research: Reporting In Vivo Experiments

Carol Kilkenny^1^, William J Browne^2^, Innes C Cuthill^3^, Michael Emerson^4^ and Douglas G Altman^5^

*^1^The National Centre for the Replacement, Refinement and Reduction of Animals in Research, London, UK, ^2^School of Veterinary Science, University of Bristol, Bristol, UK, ^3^School of Biological Sciences, University of Bristol, Bristol, UK, ^4^National Heart and Lung Institute, Imperial College London, UK, ^5^Centre for Statistics in Medicine, University of Oxford, Oxford, UK.*

|  | | ITEM | RECOMMENDATION | Section/ Paragraph |
| --- | --- | --- | --- | --- |
| 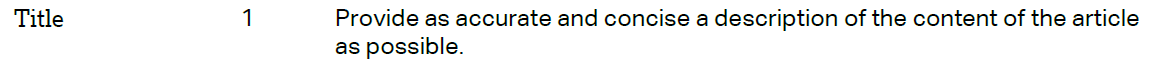 | | | Title page |  |
| 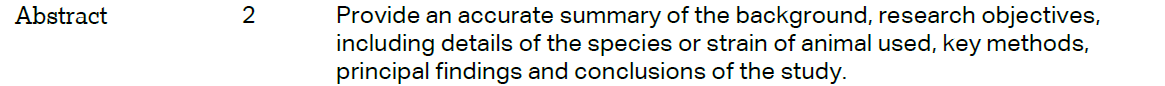 | | | Abstract |  |
| INTRODUCTION | | |  |  |
| 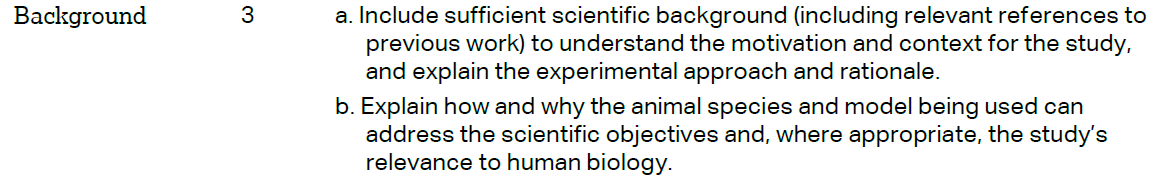 | | | Introduction  Introduction |  |
| 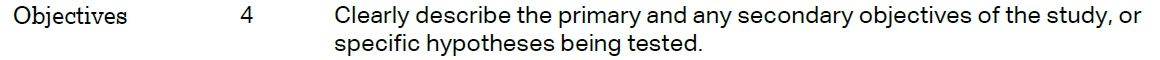 | | | Introduction |  |
| METHODS | | |  |  |
| 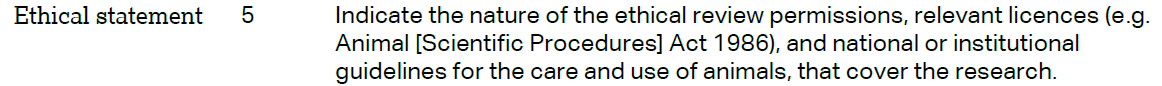 | | | Materials and Methods |  |
| 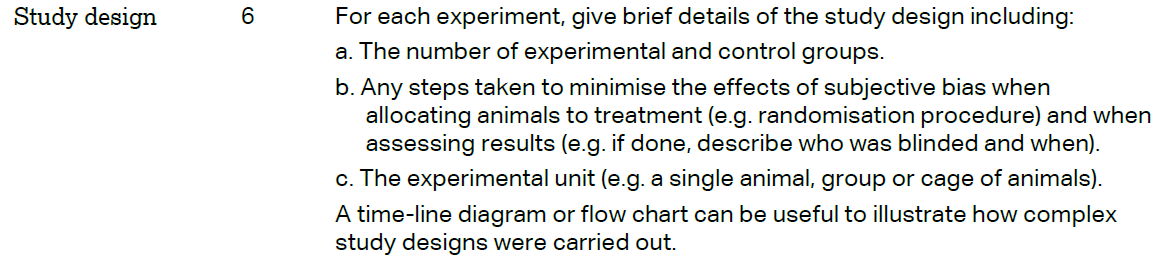 | | | Materials & Methods  Materials & Methods  Materials & Methods |  |
| 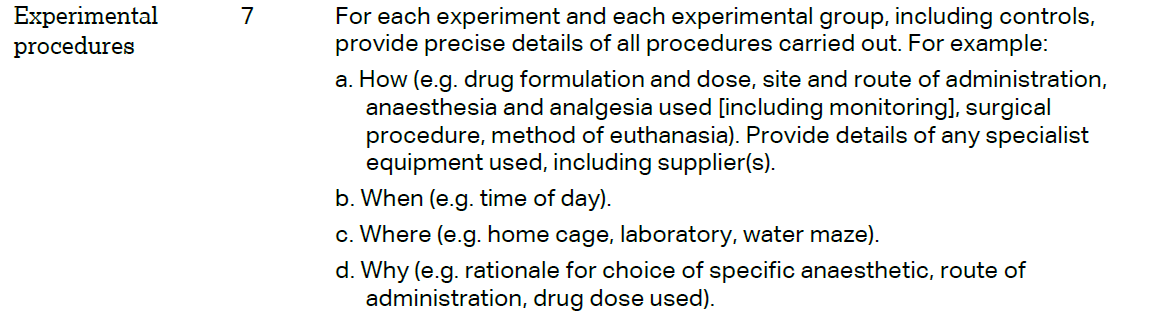 | | | Materials & Methods  Materials & Methods  Materials & Methods  Materials & Methods |  |
| 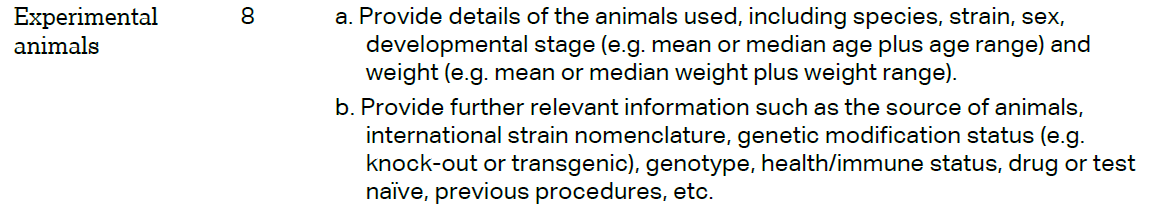 | | | Materials & Methods  Materials & Methods |  |

The ARRIVE guidelines. Originally published in *PLoS Biology*, June 2010^1^

| 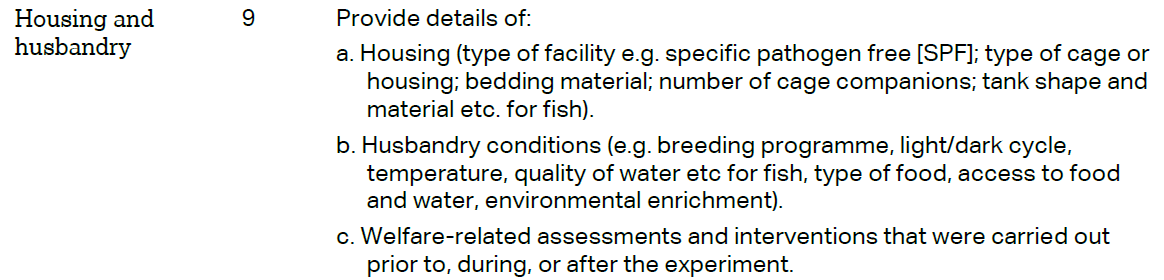 | Materials & Methods  Materials & Methods  Materials & Methods | |
| --- | --- | --- |
| 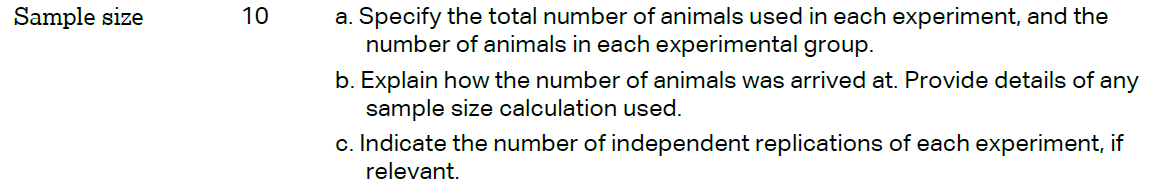 | Materials & Methods  Results  Results | |
| 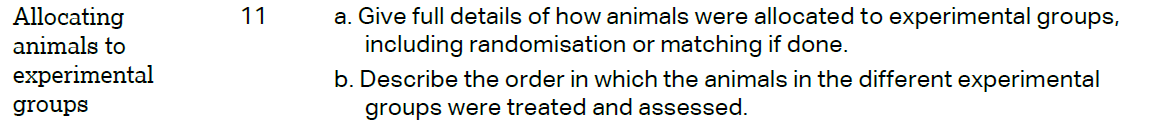 | Methods & Materials  Methods & Materials | |
| 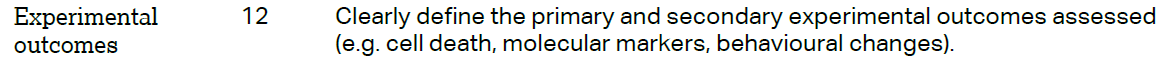 | Materials & Methods | |
| 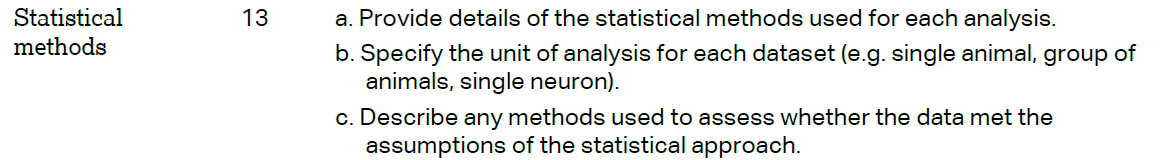 | Materials & Methods  Results  Materials & Methods | |
| RESULTS |  | |
| 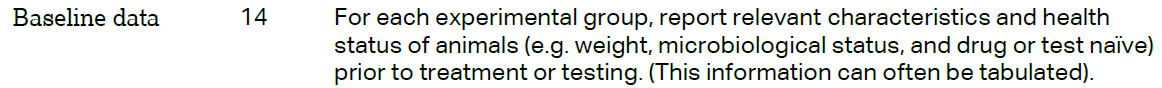 | Materials & Methods | |
| 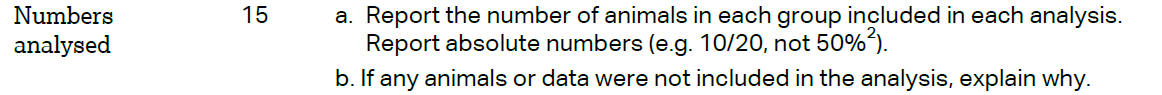 | Results | |
| 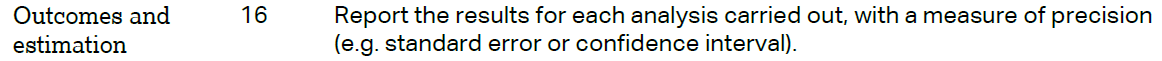 | Results | |
| 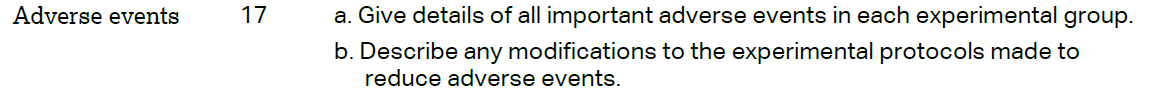 | Results | |
| DISCUSSION |  | |
| 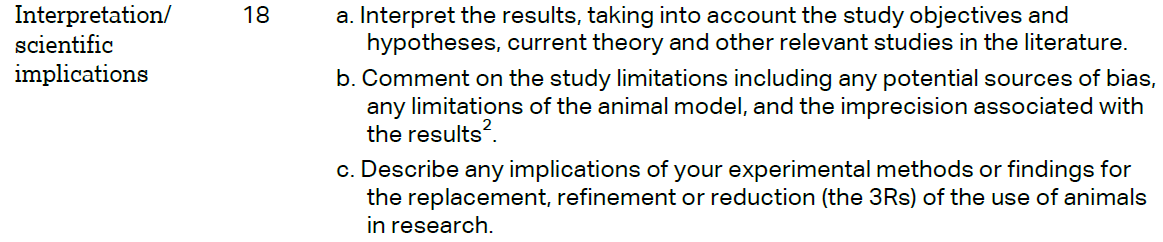 | Discussion  Discussion  N/A | |
| 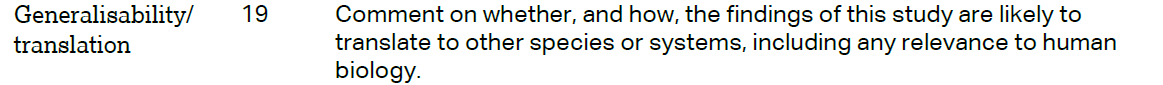 | Discussion | |
| 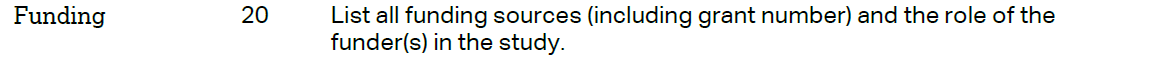 | | Acknowledgements |


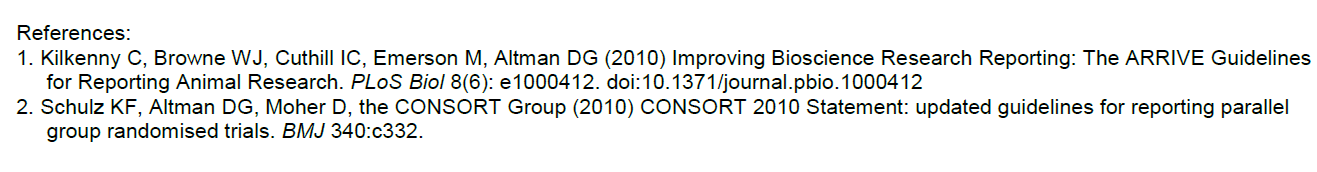

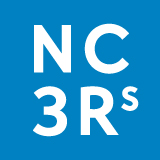

Supplement: S1 Checklist — (DOCX) [file pone.0116933.s001.docx]
