# Supplementary material for: Early Intervention for Spinal Cord Injury with Human Induced Pluripotent Stem Cells Oligodendrocyte Progenitors
Source: PLoS One. 2015 Jan 30;10(1):e0116933. doi: 10.1371/journal.pone.0116933 (PMC4311989; doi:10.1371/journal.pone.0116933)
Supplement: S1 Table — Three different iPS cell lines were used in this study: BC1, MR31 and A1-4. The sources of the these cells and the reprogramming methods used are summarized in this table. (DOCX) [file pone.0116933.s002.docx]

| CELL LINE | REPROGAMMING METHOD | SOURCE |
| --- | --- | --- |
| BC1 | EBNA1/OriP episomal vectors containing *OCT4, SOX2, KLF4, MYC, LIN28* | Adult Bone Marrow CD34+ Cells |
| MR31 | Retroviral Vector containing *OCT4, SOX2, KLF4* | IMR90 Fetal Lung Fibroblasts |
| A1-4 | Retroviral Vector containing *OCT4, SOX2, KLF4, MYC, HMGA1* | Adult Bone Marrow Mesenchymal Stem Cells |
