# Supplementary material for: Early Intervention for Spinal Cord Injury with Human Induced Pluripotent Stem Cells Oligodendrocyte Progenitors
Source: PLoS One. 2015 Jan 30;10(1):e0116933. doi: 10.1371/journal.pone.0116933 (PMC4311989; doi:10.1371/journal.pone.0116933)
Supplement: S2 Table — (DOCX) [file pone.0116933.s003.docx]

| Antibody | Manufacturer | | Catalog Number |
| --- | --- | --- | --- |
| NESTIN | Millipore | | mab5326 |
| A2B5 | Millipore | | mab312R |
| NG2 | Millipore | | ab5320 |
| PDGFR-α | Abcam | | ab61219 |
| RIP | Millipore | | mab1580 |
| O1 | Millipore | | mab344 |
| O4 | Millipore | mab345 | |
| MAG | Millipore | mab1567 | |
| MOBP | Abcam | ab66253 | |
| Human anti-Nuclei Antibody | Millipore | mab1281 | |
| MBP | Abcam | ab7349 | |
| TUJ1 | Covance | MMS-435P | |
| GFAP | Millipore | AB5804 | |
| CD68 | Abcam | AB955 | |
| OCT4 | BD Bioscience | 611203 | |
| NANOG | R&D Systems | AF1997 | |
