# Supplementary material for: Early Intervention for Spinal Cord Injury with Human Induced Pluripotent Stem Cells Oligodendrocyte Progenitors
Source: PLoS One. 2015 Jan 30;10(1):e0116933. doi: 10.1371/journal.pone.0116933 (PMC4311989; doi:10.1371/journal.pone.0116933)
Supplement: S3 Table — (DOCX) [file pone.0116933.s004.docx]

| Gene | Primer Catalog Number |
| --- | --- |
| Nanog | Hs02387400_g1 |
| Oct4 | Hs00742896_s1 |
| Sox2 | Hs01053049_s1 |
| Ng2 | Hs00426981_m1 |
| Mobp | Hs00379220_m1 |
| Mag | Hs00159000_m1 |
